# Underlying causes of Eurasian midcontinental aridity in simulations of mid‐Holocene climate

**DOI:** 10.1002/2017GL074476

**Published:** 2017-09-09

**Authors:** Patrick J. Bartlein, Sandy P. Harrison, Kenji Izumi

**Affiliations:** ^1^ Department of Geography University of Oregon Eugene Oregon USA; ^2^ Centre for Past Climate Change, School of Archaeology, Geography and Environmental Science University of Reading Reading UK; ^3^ Laboratoire de Météorologie Dynamique, IPSL, CNRS Université Pierre et Marie Curie Paris France; ^4^ Laboratoire des Sciences du Climat et de l'Environnement, CNRS‐CEA‐UVSQ Université Paris Saclay Gif‐sur‐Yvette France

**Keywords:** paleoclimate, CMIP5/PMIP3, mid‐Holocene, Eurasia, data‐model comparison

## Abstract

Climate model simulations uniformly show drier and warmer summers in the Eurasian midcontinent during the mid‐Holocene, which is not consistent with paleoenvironmental observations. The simulated climate results from a reduction in the zonal temperature gradient, which weakens westerly flow and reduces moisture flux and precipitation in the midcontinent. As a result, sensible heating is favored over evaporation and latent heating, resulting in substantial surface‐driven atmospheric warming. Thus, the discrepancy with the paleoenvironmental evidence arises initially from a problem in the simulated circulation and is exacerbated by feedback from the land surface. This region is also drier and warmer than indicated by observations in the preindustrial control simulations, and this bias arises in the same way: zonal flow and hence moisture flux into the midcontinent are too weak, and feedback from the land surface results in surface‐driven warming. These analyses suggest the need to improve those aspects of climate models that affect the strength of westerly circulation.

## Key Points

- CMIP5/PMIP3 mid‐Holocene simulations in the Eurasian midcontinent are too dry and too warm relative to paleoclimatic observations
- This mismatch is attributed to simulation of weak westerlies and moisture fluxes, amplified by surface‐energy‐balance and surface‐water‐balance feedback
- The mismatch is similar to bias in climate model simulations for the present day

## 1 Introduction

The Coupled Model Intercomparison Project Phase 5/Palaeoclimate Modelling Intercomparison Project Phase 3 (CMIP5/PMIP3) mid‐Holocene simulations show drier conditions in the Eurasian midcontinent and a significant increase in summer temperature; in contrast, paleoenvironmental data (including lake level, vegetation and isotope records, and aeolian deposits) and quantitative climate reconstructions show that the midcontinental extratropics were wetter than today and summers were cooler [*Harrison et al*., 2015]. Eurasian midcontinental aridity and warming has been a persistent feature of model simulations, already present in atmosphere‐only simulations [*Yu and Harrison*, 1996] and appearing more strongly in coupled ocean‐atmosphere simulations [e.g., *Braconnot et al*., 2007b; *Wohlfahrt et al*., 2008; *Harrison et al*., 2015] and further exacerbated by vegetation feedback [*Wohlfahrt et al*., 2004]. The consistency among multiple lines of paleoenvironmental evidence makes it unlikely that the mismatch reflects misinterpretation of the data. Regional temperature biases in the CMIP5 twentieth century simulations have been linked to biases in surface energy and water balances, with overprediction or underprediction of moisture fluxes and evapotranspiration leading to cold and warm temperature biases, respectively [*Mueller and Seneviratne*, 2014]. This suggests that discrepancies in the simulation of mid‐Holocene climates might have a similar cause. In this paper, we investigate the processes involved in midcontinental climate changes in the CMIP5/PMIP3 simulations in order to identify the underlying cause of the mismatch with observations.

## 2 Data and Methods

We have examined climate responses in the CMIP5/PMIP3 mid‐Holocene (*midHolocene*) experiments, expressed as anomalies relative to a preindustrial control (*piControl*) simulation. The *midHolocene* experiment shows the response to changes in the seasonal and latitudinal distribution in insolation 6000 years ago, with greenhouse gas concentrations at preindustrial levels (for details of the experimental design, see *Braconnot et al*. [2012]). We use outputs from 14 models (see the supporting information for details) and use the data reduction steps followed by *Harrison et al*. [2014]. Long‐term monthly averages of temperature, energy balance, and circulation variables at each model grid cell were calculated based on the last 100 years of each model simulation and bilinearly interpolated onto a common 2° × 2° grid for analysis. The “multimodel mean” maps were produced by simple averaging of model outputs. Area‐weighted averages of the key variables for each model were calculated for the Eurasian midcontinent land area between 40° to 60°N and 30° to 120°E. We exclude one model (FGOALS‐S2) from the multimodel average because it shows aberrant behavior compared to the other members of the ensemble (see the supporting information).

There are no spatially explicit reconstructions of the full set of variables analyzed here for the interval corresponding to the *piControl* simulation (circa 1850 Common Era (C.E.)). We therefore use National Centers for Environmental Prediction‐Department of Energy (NCEP‐DOE) Reanalysis 2 data (hereafter NCEP [*Kanamitsu et al*., 2002]) to investigate potential biases in the *piControl* simulations, a relatively common procedure for such evaluations [e.g., *Otterå et al*., 2009; *Wang et al*., 2010; *Perez‐Sanz et al*., 2014]. The NCEP data cover the period from 1979 to 2016, and we created monthly averages of the appropriate variables based on the period 1981 to 2010. The late twentieth/early 21st century is globally warmer than the interval around 1850 C.E., so these comparisons are indicative rather than diagnostic.

**Figure 1:**
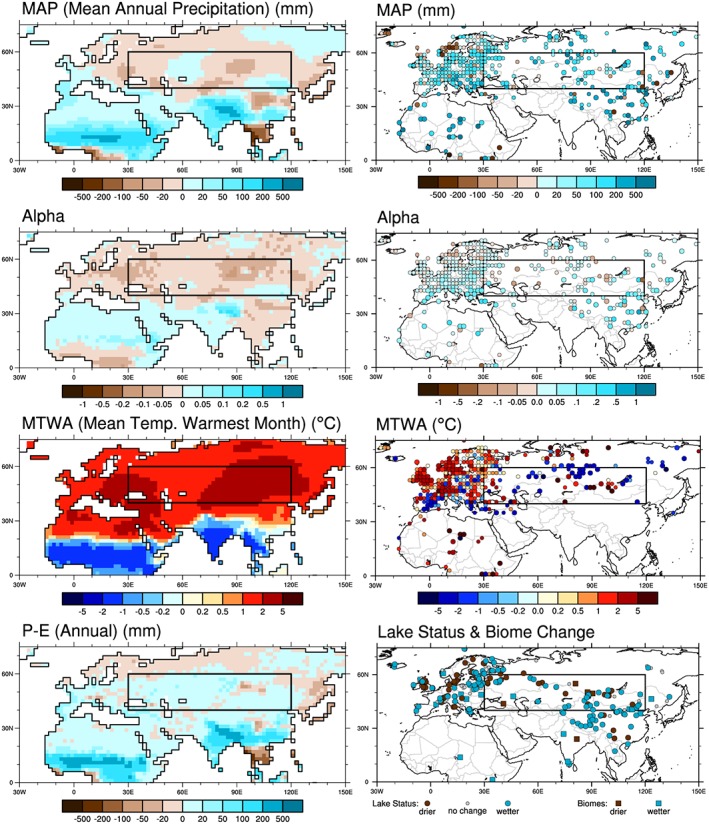
(left column) Long‐term mean differences (*midHolocene* minus *piControl*) of mean annual precipitation (MAP), the Cramer‐Prentice index of soil moisture availability (Alpha), mean temperature of the warmest month (MTWA), and precipitation minus evaporation (*P* − *E*), calculated as in *Harrison et al*. [2014], and (right column) paleoclimatic reconstructions of mid‐Holocene minus present values of MAP, Alpha, and MTWA [*Bartlein et al*., 2011], along with lake status and biome change inferred differences in moisture (see data sources in the supporting information). The region of interest here (40° to 60°N and 30° to 120°E) is indicated by the gray box in each panel.

The mid‐Holocene (MH) has been a major focus for paleoenvironmental and paleoclimate synthesis. We use data from the Global Lake Status Database (GLSDB [*Tarasov et al*., 1994; *Yu and Harrison*, 1995; *Harrison et al*., 1996; *Tarasov et al*., 1996; *Yu et al*., 2001]) and the BIOME 6000 [*Harrison et al*., 2017] database to document regional water balance and vegetation cover (Figure 1). The GLSDB provides changes in lake status relative to present at individual lake sites on the radiocarbon timescale; the age models for each site were converted to calendar years (using the INTCAL13 calibration [*Reimer et al*., 2013]) in order to select information for 6000 years B.P. Lake status is an index of the water balance (precipitation minus evaporation) over the lake and its catchment. Lake histories produced since the publication of the GLSDB have also been compiled and are included in our analyses—see the supporting information for details [*Bird et al*., 2014; *Boomer et al*., 2000; *Chen et al*., 2008; *Chawchai et al*., 2013; *Chen et al*., 2003; *Ferronskii et al*., 2003; *Fowell et al*., 2003; *Gasse et al*., 1996; *Grunert et al*., 2000; *Kong et al*., 2007; *Heinecke et al*., 2016; *Herzschuh et al*., 2004; *Hodell et al*., 1999; *Huang et al*., 2009, 2014; *Jiang et al*., 2007; *Jiang and Liu*, 2007; *Li and Morrill*, 2010; *Li et al*., 2011, 2014a, 2014b; *Long et al*., 2012; *Madsen et al*., 2008; *Mathis et al*., 2012; *Mingram et al*., 2004; *Morinaga et al*., 1993; *Morrill et al*., 2006; *Pan et al*., 2012; *Peck et al*., 2002; *Penny et al*., 1996; *Prokopenko et al*., 2007; *Rades et al*., 2015; *Ricketts et al*., 2001; *Schwanghart et al*., 2008; *Shen et al*., 2005; *Sheng et al*., 2017; *Wang et al*., 2017; *Wang and Ji*, 1995; *Wünnemann et al*., 2003, 2006; *Xiao et al*., 2008; *Yang et al*., 2015; *Zhang et al*., 2004, 2014; *Zhao et al*., 2007, 2010, 2013; *Zhou et al*., 2015]. BIOME 6000 provides pollen‐based reconstructions of vegetation for the MH [*Prentice et al*., 2000; *Bigelow et al*., 2003], and the reconstructions for Eurasia have been updated by *Binney et al*. [2017]. Changes between forest and nonforest vegetation closely reflect water availability, except when CO_2_ is low [*Prentice et al*., 2017], and thus provide a cross check on inferences drawn from lake status. Pollen data have also been widely used to reconstruct climate variables quantitatively. Here we use the compilation of *Bartlein et al*. [2011], which provides gridded (2° × 2°) reconstructions of six climate variables expressed as anomalies from present‐day observations [*New et al*., 2002]; we use mean temperature of the warmest month (MTWA), mean annual precipitation (MAP), and the Cramer and Prentice (1988) index of soil moisture availability (*α*).

## 3 Results and Discussion

There is substantial agreement in the spatial and seasonal expression of the sign of change in the simulated climate variables across individual models (see the supporting information), which allows us to focus here on the multimodel average responses. Mean annual precipitation (MAP) is reduced very little (the multimodel area‐weighted average is −0.02 mm d^−1^ in the belt between 40° and 60°N) over the Eurasian midcontinent in the CMIP5/PMIP3 *midHolocene* simulations compared to the *piControl* (Figure 1), and this translates into a similarly small reduction in both runoff (*P* − *E*) and soil moisture (as measured by *α*). Drier (than *piControl*) conditions are most marked in the belt between 40° and 60°N. Summer temperature is significantly higher than in the *piControl* (Figure 1): the multimodel area‐weighted average increase in MTWA in the belt between 40° and 60°N is +2.1°C. In contrast, many lakes across the region were higher than today and vegetation data indicate the persistence or even expansion of forests (Figure 1), both implying that the midcontinental region was wetter than today. Paleoclimate reconstructions indicate that both MAP and *α* were higher than today (Figure 1); the area‐weighted average regional increase in precipitation is 120 ± 21 mm yr^−1^, and the area‐weighted increase in *α* is 0.024 ± 0.010 (where the uncertainties are the reconstruction standard errors) [see *Bartlein et al*., 2011]. The reconstructions of MTWA are more heterogeneous (Figure 1), with some differences between simulated and reconstructed summer temperatures of up to 4°C at individual sites; however, reconstructed summer temperatures averaged across the whole region are similar to those of today (−0.06 ± 0.48°C). The *midHolocene* simulations therefore appear to be too dry and too warm relative to the paleoclimatic observations.

Although generally dry, the region of interest has a distinct summer wet season, when soil moisture is replenished by higher precipitation provided by increased moisture flux from westerly sources (see the supporting information), and therefore, changes in the strength of the westerlies and the moisture flux are key for understanding changes in moisture. Moisture changes in turn contribute to temperature changes through the partitioning of net radiation into latent as opposed to sensible and substrate heating.

**Figure 2:**
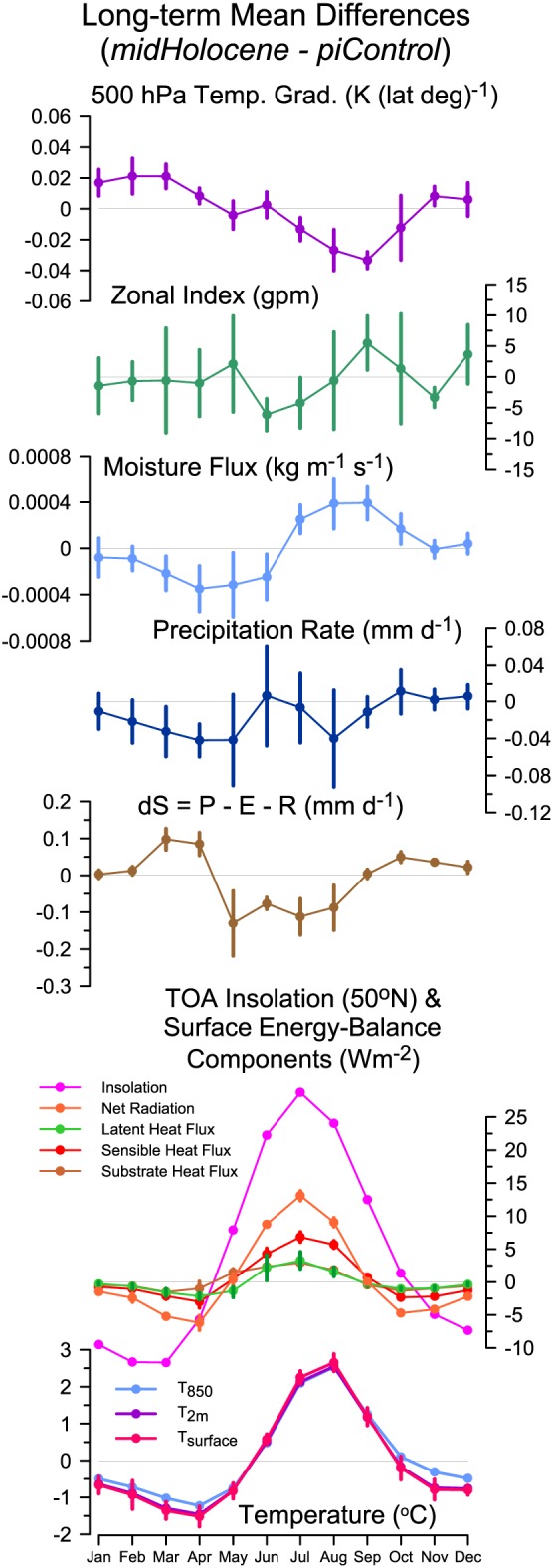
Annual cycles of *midHolocene* minus *piControl* differences. The values shown are area‐weighted averages for grid points in the region 40° to 60°N and 30° to 120°E for the multimodel mean. The vertical bars extent to plus or minus one median absolute deviation (MAD, a robust measure of model spread) either side of the average. For clarity among the temperature curves, these are shown only for surface (or “skin”) temperature (*T*_surface_).

As a result of the MH changes in the seasonal and latitudinal patterns of insolation consequent on the change in orbital forcing (and in particular the substantial increase in summer), the latitudinal temperature gradient in the Northern Hemisphere was less steep than today during the winter (November through April) but steeper than today during summer (July to September; Figure 2). The 500 hPa zonal index (a measure of the strength of westerly flow, calculated as the horizontal geopotential height gradient or difference between 40° and 60°N, over the longitudinal span 30° to 120°E), shows considerable month‐to‐month and model‐to‐model variability, but moisture flux into the midcontinent is clearly reduced relative to *piControl* over the first half of the year. Precipitation is lower than *piControl* through May and little different afterward (Figure 2). As a result of the reduction in spring precipitation, soil moisture is not replenished and is lower than *piControl* throughout the summer (Figure 2). Although this reduction in soil moisture would ordinarily be expected to limit evapotranspiration, evapotranspiration (latent heat flux) is higher in the *midHolocene* simulation compared to *piControl* because of the higher summer insolation. The excess energy is preferentially used for sensible heating, resulting in much larger increases in sensible than latent heat: in July, 7.1 versus 3.2 W m^−2^ respectively. The increase in sensible heat flux results in increased surface and atmospheric temperature. Comparison of the changes in air temperature at different elevations (surface, 2 m, 850 hPa) confirms that summer warming is a surface‐driven phenomenon (see the supporting information). The effect of the positive energy balance feedback on temperature is strong enough to overwhelm the discernable, though small, intermodel variability in circulation and moisture.

Reconciliation of the simulated and reconstructed MH temperatures in Eurasia would require increased delivery of precipitation into the midcontinent to offset the impact of the strong positive energy balance feedback. This would require the simulated westerly flow and moisture flux to be stronger. However, the simulated circulation in the *piControl* would also need to be stronger to be consistent with the impact of orbital changes on the change in latitudinal temperature gradients between the *midHolocene* and *piControl* simulations. Evaluation of the *piControl* simulations against observations can show whether this is feasible, i.e., whether there is evidence to suggest that the simulated circulation is weaker than observed and this contributes to obvious biases in surface climates in the midcontinent.

**Figure 3:**
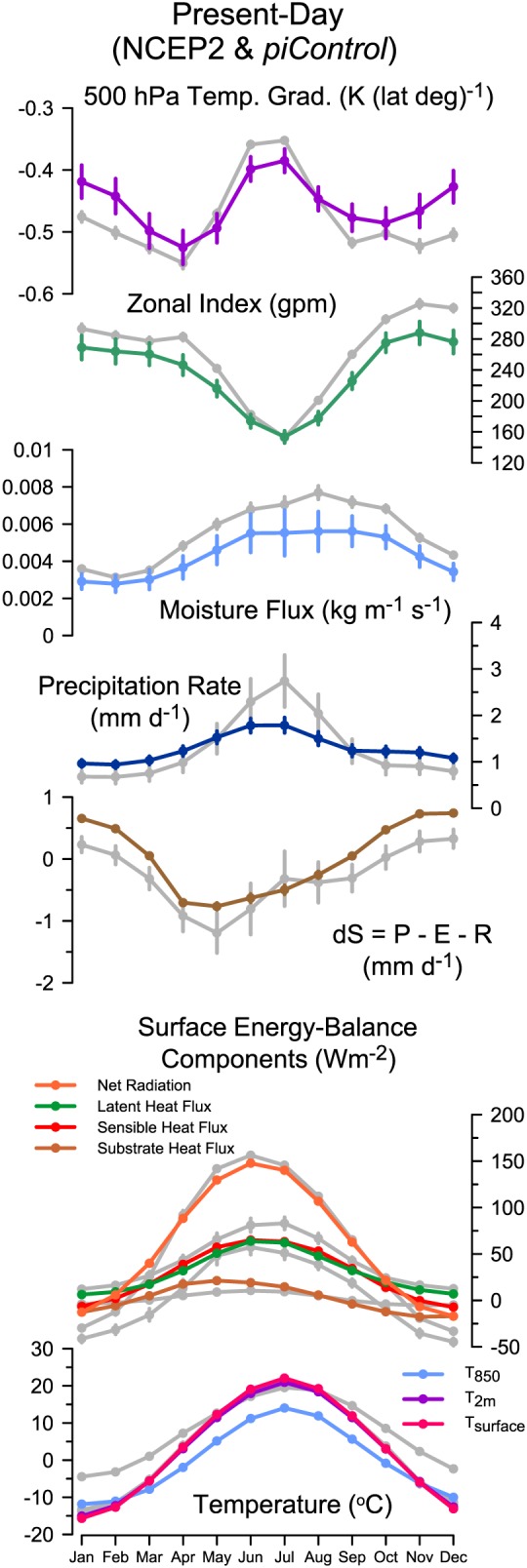
Annual cycles of *piControl* and NCEP reanalysis long‐term averages. The values shown are area‐weighted averages for grid points in the region 40° to 60°N and 30° to 120°E for the multimodel mean. The NCEP values are plotted in gray for each variable. The uncertainties shown extend to plus or minus two standard errors of the mean, based on the interannual variability of the individual grid point values.

The latitudinal temperature gradient in the *piControl* simulations (Figure 3) is weaker than shown by NCEP during most of the year, though slightly stronger than the reanalysis in June and July. The 500 hPa zonal index mirrors this, being similar to NCEP in summer and stronger than NCEP in winter. The simulated strength of the westerly flow is weaker than in the reanalysis, and thus, moisture flux is reduced. The *piControl*‐simulated precipitation is higher than observed in winter, although the amount is very small (e.g., simulated rate 0.96 mm d^−1^ compared to 0.68 mm d^−1^ in January), and much lower than observed in summer (e.g., simulated rate 1.79 mm d^−1^ compared to 2.74 mm d^−1^ in July). As in the *midHolocene* case, the dry bias is exacerbated by the energy balance feedback: soil moisture is generally lower than in the reanalysis throughout the year, latent heat is lower and sensible heating higher than in the reanalysis. The mechanisms that give rise to biases in surface climates in the *midHolocene* simulations are thus operating in the *piControl*, and the circulation‐related variables are indeed weaker than in the reanalysis. This discrepancy is particularly noteworthy because the *piControl* climate is globally colder than the twentieth/21st centuries and the latitudinal temperature gradient steeper, and so a priori [*Rind*, 1998], the simulated circulation should be stronger than the NCEP reanalysis. These analyses suggest that removing the circulation bias under modern‐day conditions would lead to a better simulation of MH climates in midcontinental Eurasia.

## 4 Conclusions and Implications

We have shown that the erroneous simulation of warmer and drier summers in Eurasia during the MH (relative to present) is ultimately caused by the weaker westerly circulation, which results in low precipitation in early summer and is a precondition for the operation of strong positive energy balance feedbacks in driving simulated temperature increases. Data‐model comparisons of regional paleoclimates have often focused on model benchmarking, i.e., diagnosis of the quantitative mismatch between simulated and observed climates [e.g., *Braconnot et al*., 2007a; *Wohlfahrt et al*., 2008; *Otto‐Bliesner et al*., 2009; *Zhang et al*., 2010; *Braconnot et al*., 2012; *Harrison et al*., 2014; *Schmidt et al*., 2014; *Mauri et al*., 2014; *Perez‐Sanz et al*., 2014]. Diagnosis of the processes that contribute to model biases is less common but important in order to identify ways in which the current generation of climate models could be improved. Several papers have shown that thermodynamic responses in paleoclimate simulations are reasonable [e.g., *Izumi et al*., 2013, 2015], implying that regional data‐model mismatches are more likely to be due to problems with dynamics; our results are consistent with this. Explanations for the poor simulation of European climate during the MH have also invoked circulation as a major cause of the problem [*Bonfils et al*., 2004; *Mauri et al*., 2014]. However, in recent assessments [e.g., *Randall et al*., 2007; *Flato et al*., 2013] of the ability of state‐of‐the‐art models to capture modern climates, however, the major focus has been on storm track variability and extremes rather than evaluation of basic circulation patterns. Our analyses suggest that even the basic circulation patterns are poorly captured.

In our analyses, we have compared reconstructions of surface climates to model outputs. We have not attempted to diagnose atmospheric circulation directly. Although there have been some attempts to reconstruct paleocirculation [e.g., *Kohfeld et al*., 2013], direct evidence for wind pathways, direction, and strength is limited. In general, the aeolian landforms that provide direct evidence are difficult to date because of their sedimentary composition. Furthermore, it is rarely possible to determine whether the formation of these landforms reflects long‐term mean wind conditions or sporadic and atypical winds. Oxygen isotope records may ultimately hold more promise for the diagnosis of circulation changes [e.g., *Herold and Lohmann*, 2009; *Caley et al*., 2014; *Dietrich et al*., 2013].

The long‐term average differences in both circulation‐related and surface energy balance variables, both seasonally and in their spatial patterning, are remarkably similar across the suite of CMIP5/PMIP3 *midHolocene* simulations. Such robustness in the simulated climate is often taken as a sign that the signal is correct, for example, in the interpretation of future regional climate changes [e.g., *Ciscar et al*., 2011]. Indeed, the use of the multimodel median as a basis for model ranking [e.g., *Flato et al*., 2013] is predicated on the idea that similarity to other models is a criterion of value. There are multiple instances where the paleorecord shows that a robust signal is nevertheless unrealistic [*Harrison et al*., 2015]. However, the example of midcontinental Eurasia is an extreme case, because the simulations and the observations differ not only in magnitude but also in sign.

In conclusion, the simulation of circulation needs to be improved in order to simulate midcontinental Eurasian surface climates better, both in the mid‐Holocene and under modern conditions. It is generally assumed that increasing model resolution, through improving the representation of topography and land‐sea geography, will improve the simulation of circulation and atmospheric dynamics [*Jung et al*., 2012]. The paleorecord from the Eurasian midcontinent could play a crucial role in testing whether this is true.

## Supporting information



Supporting Information S1

Data Set S1
