# Supplementary material for: Underlying causes of Eurasian midcontinental aridity in simulations of mid‐Holocene climate
Source: Geophys Res Lett. 2017 Sep 9;44(17):9020–8. doi: 10.1002/2017GL074476 (PMC5661745; doi:10.1002/2017GL074476)
Supplement: Supplementary file 1 — Supporting Information S1 [file GRL-44-9020-s001.docx]

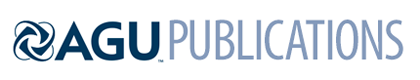


*Geophysical Research Letters*

**Underlying causes of Eurasian mid-continental aridity in simulations of
mid-Holocene climate**

Patrick J. Bartlein^1^, Sandy P. Harrison^2^ and Kenji Izumi^1,3,4^

^1^ Department of Geography, University of Oregon, Eugene, Oregon, USA

^2^ Centre for Past Climate Change, School of Archaeology, Geography and Environmental Science, University of Reading, Reading, UK

^3^ Laboratoire de Météorologie Dynamique, IPSL, CNRS, Université Pierre et Marie Curie,
Paris 75005, France

^4^ Laboratoire des Sciences du Climat et de l’Environnement, CNRS-CEA-UVSQ,
Université Paris-Saclay, Gif-sur-Yvette cedex 91191, France

**Contents of this file**

Text S1 to S5

Figures S1 to S31

Tables S1 to S2

**Additional Supporting Information (Files uploaded separately)**

**Data Set S1.** Lake status at 6000 cal yr BP compared to present day.

**Introduction**

This supplemental file contains text components that describe:

- the data sources used here, including the paleoclimatic simulations and observations, and their URLs (Text S1);
- in brief, the data-reduction and analysis applied to the climate-model output, which is described in detail in *Harrison et al.* [2014] (Text S2);
- the analysis of the paleoclimatic reconstructions (Text S3);
- the calculation of a diagnostic soil-moisture variable that mitigates the heterogeneous representation of soil moisture among models (Text S4); and
- the supplemental figures (Text S5)

Supplemental figures are included that illustrate:

- the mismatch between the simulations and paleoclimatic reconstructions using boxplots (Figure S1);
- plots of the annual cycles of the long-term means of the variables used in the analyses for the *piControl* and *midHolocene* simulations, as well as their long-term mean differences (Figures S12 to S15).
- representative maps of individual variables that illustrate the robustness among models of the simulated long-term differences between the *midHolocene* and *piControl* simulations (Figures S16 to S18);
- maps of the simulated long-term differences between the *midHolocene* and *piControl* simulations for the variables used in the analyses here (Figures S19 to S30);

Two tables are provided that list the variables used here and climate-model simulations.

A supplemental data set (ds01.pdf) contains the lake-status reconstructions for Eurasia.

**Text S1. Data Sources**

Data used in the analyses here include climate-model output from the CMIP5/PMIP3 archive, along with paleoclimatic observations and reconstructions. All data are available online, as follows:

- Climate model output was obtained from the Earth System Grid Federation (ESGF) node at <https://esgf-node.llnl.gov>. The analyses and figures are based on data archived at ESFG on December 2015. Individual models were selected based on the availability of a suite of atmospheric circulation, surface energy- and water-balance, and surface-climate variables for both the *midHolocene* and *piControl* CMIP5 simulations. The variables are listed in Table S1, and the thirteen models chosen are listed in Table S2. Further descriptions of the individual models can be found in *Harrison et al.* [2014].
- Present-day climate was illustrated using data from the NCEP-DOE Reanalysis 2 data set provided by the National Oceanic and Atmospheric Administration, Earth System Research Laboratory, Physical Sciences Division, Boulder, Colorado, USA, from their Web site at <http://www.esrl.noaa.gov/psd/>.
- Paleoclimate reconstructions from pollen data were those from *Bartlein et al.* [2011]. These data can be downloaded from the NOAA National Climatic Data Center at <https://www.ncdc.noaa.gov/paleo/study/9897>.
- Biome data used here include those from a) the BIOME 6000 consolidated data set [*Harrison*, 2017] (available at <http://researchdata.reading.ac.uk/99/>, and b) *Binney et al.* [2017], available at <http://eprints.soton.ac.uk/id/eprint/403426>.
- Lake-status data. Lake-status reconstructions for the Eurasian region are provided in the supplementary data set ds01.pdf, and include revised information from the Global Lake Status Database <http://www.bridge.bris.ac.uk/projects/GLSDB>.
- We also used the CRU CL 2.0 1961-1990 10-minute long-term mean data (resampled to 0.5 degrees) *New et al.* [2002] as a basis for calculating bioclimatic variables (see Text S2) using the climate-model output. These data are available at <https://crudata.uea.ac.uk/cru/data/hrg/tmc/>.

**Text S2. Data Reduction and Analysis of Climate-Model Output**

The data reduction and analysis of the climate-model output involved the following steps:

1. calculation of long-term means and standard deviations on the native GCM grids using the last 100 years of each simulation,
2. calculation of long-term mean differences, *midHolocene* minus *piControl*,
3. regridding of the long-term means and long-term mean differences onto a common 2-degree (latitude by longitude) grid,
4. calculation of multi-model mean values of the long-term means and long-term mean differences using all models (except FGOALS-s2).

Four variables that are not explicitly included in the CMIP5 archive were calculated as follows:

1. The latitudinal temperature gradient at the 500 hPa level across the region was calculated as the temperature difference between grid-cell averages at 40° and 60° N (over the longitudinal span 30° to 120° E).
2. The 500 hPa zonal index (a measure of the strength of westerly flow, was calculated as the horizontal geopotential height gradient (difference) between 40° and 60° N, over the longitudinal span 30° to 120° E).
3. Moisture flux was calculated as specific humidity times the vector wind, integrated from surface to the top of the atmosphere.
4. The change in soil moisture, dS is described in section Text S4 below.

We also calculated surface evaporation rate for the NCEP 2 data as *evspsbl = L_v_*lhtfl*, where *L_v_* is the latent heat of vaporization and *lhtfl* is the latent-heat flux.

For the bioclimatic variables (MAP, Alpha, MTWA and P-E) that appear on Fig. 1, the long-term mean differences were applied to the CRU CL 2.0 1961-1990 long-term mean data. This approach allows the “high-resolution” spatial variability of climate to be included in the bioclimatic variables. Alpha was calculated using the approach of *Cramer and Prentice* [1988]. Full details of the data-reduction and analysis of the climate-model output can be found in the Supplementary Material of *Harrison et al.* [2014].

There are multiple ways of expressing “model uncertainty”, and the optimal approach depends on context. In Figure 2 here (illustrating the long-term mean differences between the *midHolocene* and *piControl* simulations), we were concerned with displaying the consistency among models, in order to motivate the use of the multi-model mean to summarize the annual cycles of the simulations and their differences. We selected the median absolute deviation (MAD) to illustrate model “spread” in Fig. 2, because it is a robust measure of variability, influenced less by outliers or unusual points than the standard deviation. In Fig. 3, we were concerned with comparing the temporal variability of the multimodel mean of the *piControl* simulation with the NCEP 2 reanalysis data (i.e. their variability over time in the present day), and the appropriate choice here is the standard error of the mean.

Median absolute deviations in Fig. 2 were calculated in the usual way, as *MAD* = median( |*X_i_* – median(*X*)|), and the standard errors of the long-term means in Fig. 3 were calculated using an area-weighted average of the standard deviations of the 100 years (CMIP5) or 30 years (NCEP 2) of time-series data.

**Text S3. Paleoclimatic reconstructions**

Uncertainties in the paleoclimatic reconstructions of *Bartlein et al.* [2011] are the published pooled reconstruction uncertainties, and are illustrated in Fig. 1 by symbol sizes: larger symbols are used to indicate reconstructed long-term mean differences that exceed twice the pooled reconstruction uncertainties. See *Bartlein et al.* [2011] for discussion.

**Text S4. Soil-moisture diagnosis**

The CMIP5 models represent soil moisture in heterogeneous ways, related to the specific definitions of the number of soil layers, overall column depth, and other variables in individual models. As a result, it is impossible to compare the long-term means (or long-term mean differences of the individual models); there is much more model-to-model variability in the long-term means than there is within the year or between the *midHolocene* and *piControl* simulations (see Fig. S14). Consequently, we developed a new variable, the change in soil moisture (dS) calculated as precipitation minus evaporation and runoff (Fig. S6). The change in soil moisture varies more coherently among the models, and the multi-model mean of the CMIP5 *piControl* values compares favorably with values calculated with the NCEP 2 data, as do the components of this variable (precipitation (Fig. S5), evaporation, derived from latent heat flux (Fig. S8) and surface runoff (Fig. 15), consistent, however, with the biases between the CMIP5 *piControl* simulations and the NCEP 2 data discussed in the main text.

**Text S5. Supplemental Figures**

There are four types of supplemental figures. Fig. S1 shows boxplots of reconstructed and simulated bioclimatic variables (MAP, Alpha, and MTWA), constructed as in *Harrison et al.* [2014]. The plots show the median (center of each) box, the interquartile range (the 25^th^ and 75 percentiles, which define the height of the box) and the 5^th^ and 95^th^ percentiles (with define the whiskers) of the reconstructions (in gray), and the CMIP5/PMIP3 simulations, interpolated to the location of the reconstructions (see *Harrison et al.* [2014] for further elaboration of the methods).

Figure. S2 to S15 show the annual cycles of the key variables discussed here, including the long-term means of the *piControl* and *midHolocene* simulations, and the long-term mean differences between the two. To assess the present-day bias in the models, data from the NCEP 2 reanalysis is shown in red on the *piControl* panels (i.e. 1981-2010 long-term means). The specific variable names in the CMIP5 archive and NCEP 2 reanalysis data set are given in table S1. The boundary conditions for the piControl simulation represent approximate 1850 CE conditions, and so it might be expected that the NCEP 2 reanalysis data should be warmer in the region of interest (between 40° to 60° N and 30° to 120° E). However, this is not the case, near-surface air temperatures (tas) are nearly identical, while surface or “skin” temperatures in the CMIP5/PMIP3 simulations are higher than in the NCEP 2 renalysis data. Note that outputs from FGOALS-S2 are shown in these plots, although the model is not used in the calculation of the multi-model ensemble because of its anomalous behavior.

Figures S16 to S18 are maps of long-term mean differences for a selection of variables (near-surface air temperature (Fig. S15), precipitation (Fig. S16) and mean sea-level pressure (Fig. S17)) for the individual models and multi-model mean for the month of August, to illustrate the low model-to-model variability in the CMIP5/PMIP3 simulations, which motivates our use of the multi-model mean to illustrate the *midHolocene* and *piControl* simulations and their differences. Note that outputs from FGOALS-S2 are shown in these plots, although the model is not used in the calculation of the multi-model ensemble because of its anomalous behavior.

Figures S19 to S30 are maps of the long-term mean difference for the key variables that underlie Fig. 2 in the main text. Figure S31 is an alternative version of Fig. 2 in the main text that shows the medians of multi-model long-term mean differences as black diamonds. The choice of either the mean or median long-term mean difference does not change the interpretation of the data shown on this figure.

**Figure S1.** Boxplots of paleoclimatic reconstructions (gray boxes) and of CMIP5/PMIP3 simulations, including ocean-atmosphere (OA), or ocean-atmosphere-carbon cycle (OAC) component models. Model-output values were sampled at the locations of the paleoclimatic reconstructions.

**Figure S2.** 500 hPa latitudinal temperature gradients, *piControl* and *midHolocene* long-term means and *midHolocene* minus *piControl* long-term mean differences. Multi-model mean values are shown in black and NCEP 2 data are shown in red. Figure legend for individual models follows Fig. S15.

**Figure S3.** 500 hPa zonal index. Legend as in Fig. S2.

**Figure S4.** Area-weighted averages of vertically integrated moisture flux (qmag). Legend as in Fig. S2.

**Figure S5.** Area-weighted averages of precipitation rate (pre). Legend as in Fig. S2.

**Figure S6.** Area-weighted averages of changes in soil moisture (dS). Legend as in Fig. S2.

**Figure S7.** Area-weighted averages of net radiation (netrad). Legend as in Fig. S2.

**Figure S8.** Area-weighted averages of latent heat flux (hfls). Legend as in Fig. S2.

**Figure S9.** Area-weighted averages of sensible heat flux (hfls). Legend as in Fig. S2.

**Figure S10.** Area-weighted averages of substrate heat flux (hflsub). Legend as in Fig. S2.

**Figure S11.** Area-weighted averages of surface (“skin”) temperature (ts). Legend as in Fig. S2.

**Figure S12.** Area-weighted averages of near-surface (2-m) air temperature (tas) Legend as in Fig. S2.

**Figure S13.** Area-weighted averages of 850 hPa temperature (ta850). Legend as in Fig. S2.

**Figure S14.** Area-weighted averages of soil moisture (mrso). Legend as in Fig. S2, but no NCEP 2 data are plotted for this variable, because there is no comparable variable. Note the varying y-axis scale on the long-term mean difference panel.

**Figure S15.** Area-weighted averages of runoff (mrros). Legend as in Fig. S2.

Legend for Figs S2 through S15:

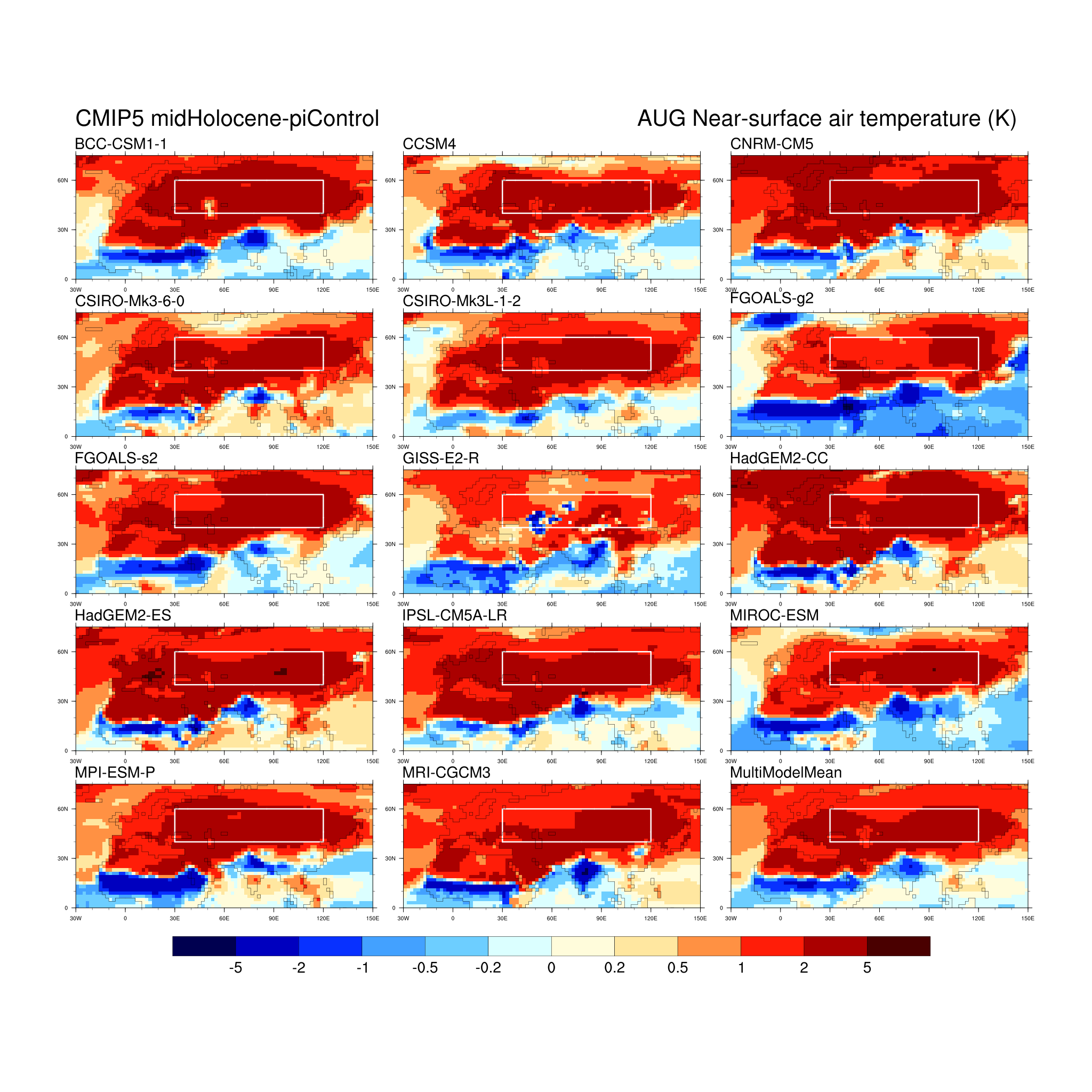


**Figure S16.** Individual model *midHolocene* minus *piControl* long-term mean differences in near-surface air temperature (tas) for August, illustrating the robustness of the simulations in the region of interest in this paper (white box).


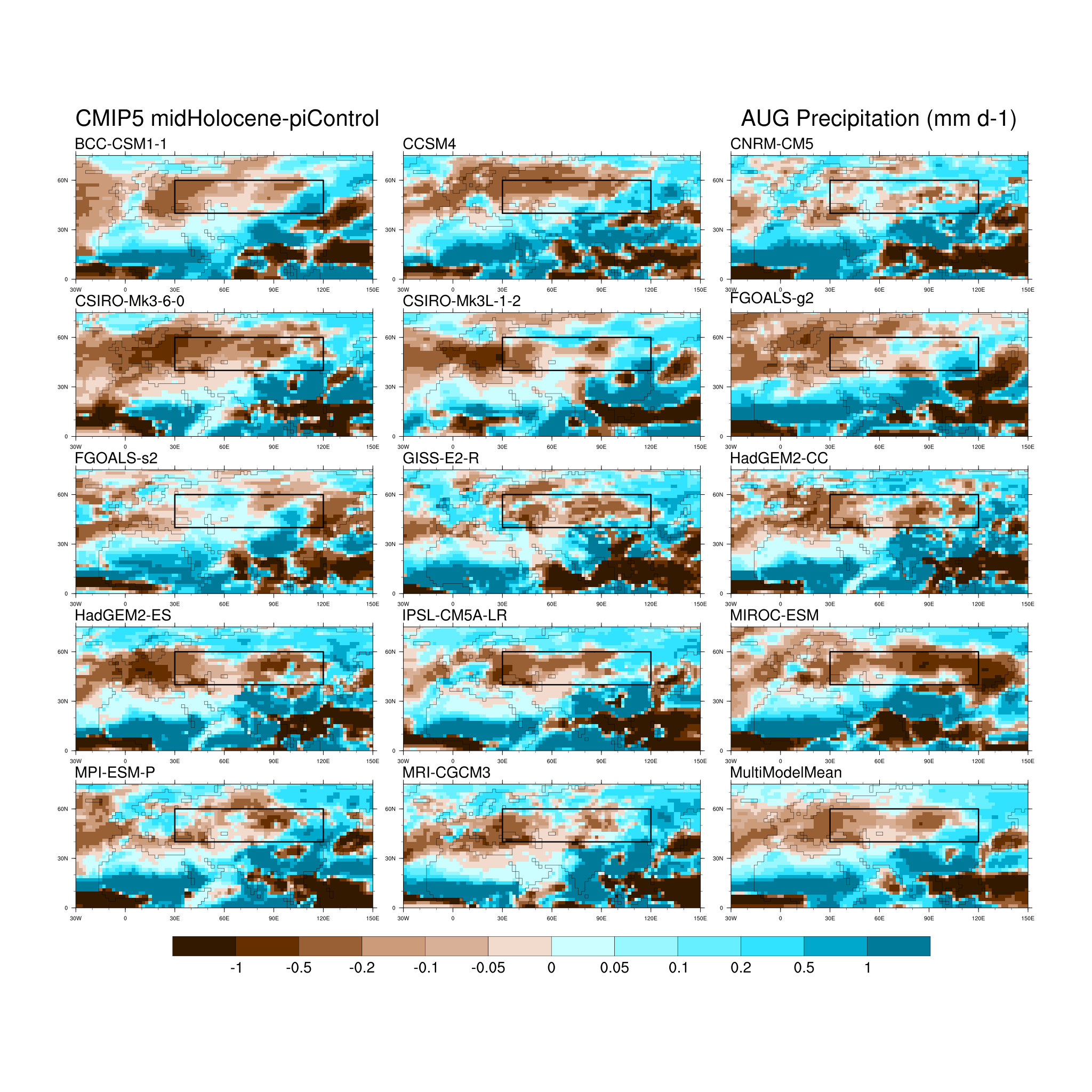


**Figure S17.** Individual model *midHolocene* minus *piControl* long-term mean differences in precipitation rate (pre) for August, illustrating the robustness of the simulations in the region of interest in this paper (black box).


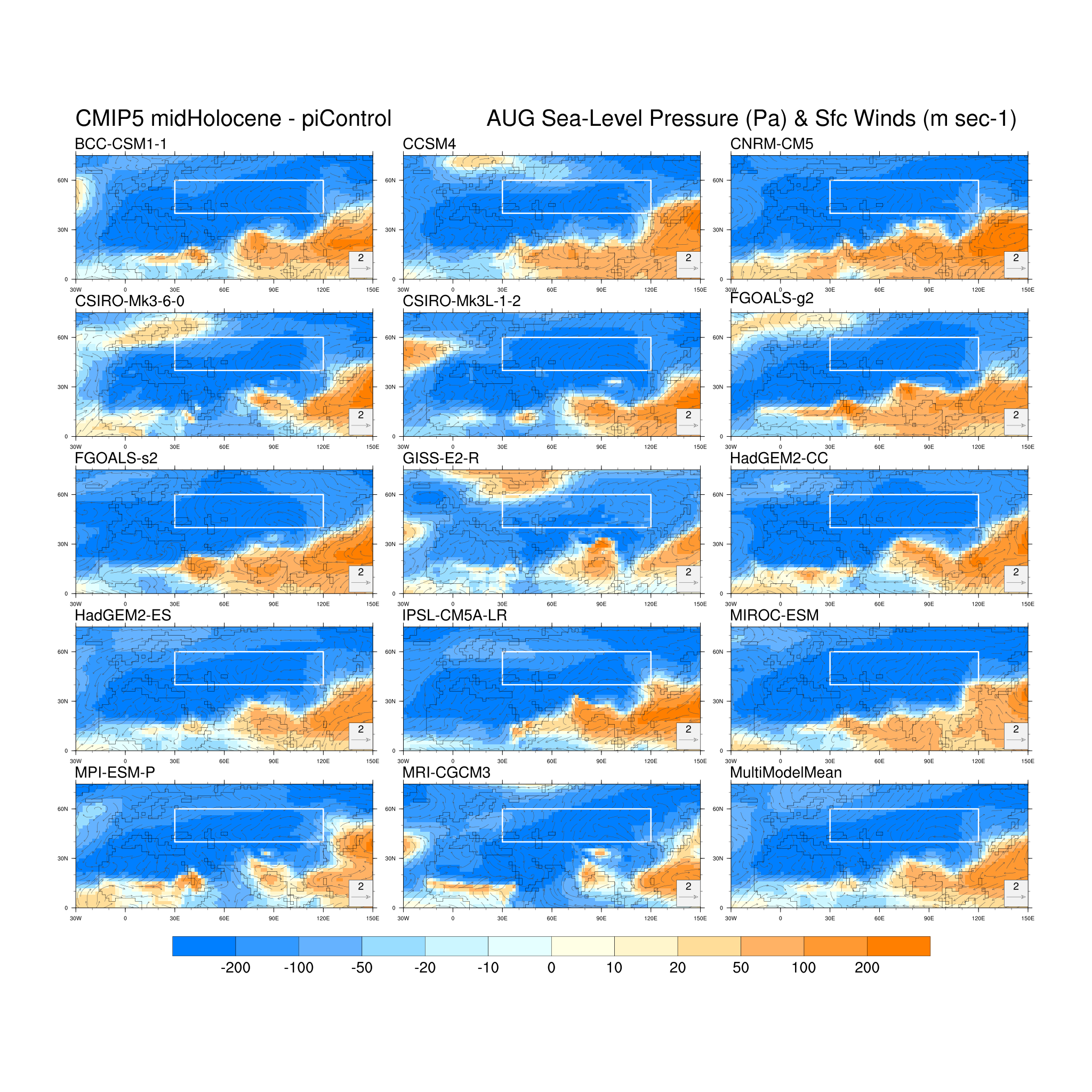


**Figure S18.** Individual model *midHolocene* minus *piControl* long-term mean differences in mean sea-level pressure (psl) and surface winds for August, illustrating the robustness of the simulations in the region of interest in this paper (white box).


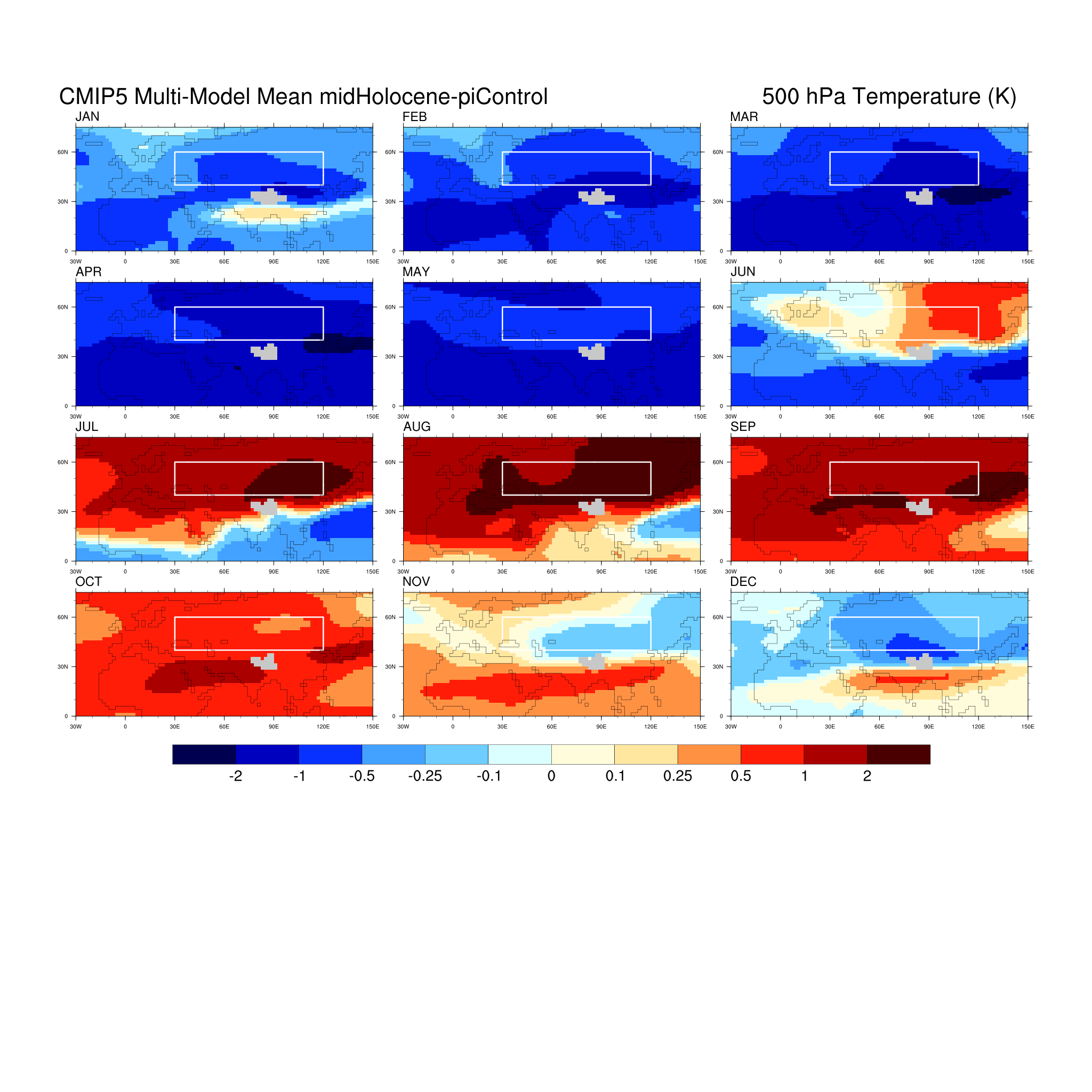


**Figure S19.** *midHolocene* minus *piControl* long-term mean differences in 500 hPa temperature (ta500). Gray areas indicate regions where the land surface is above the 500 hPa level. The region of interest in this paper is shown by the white box.


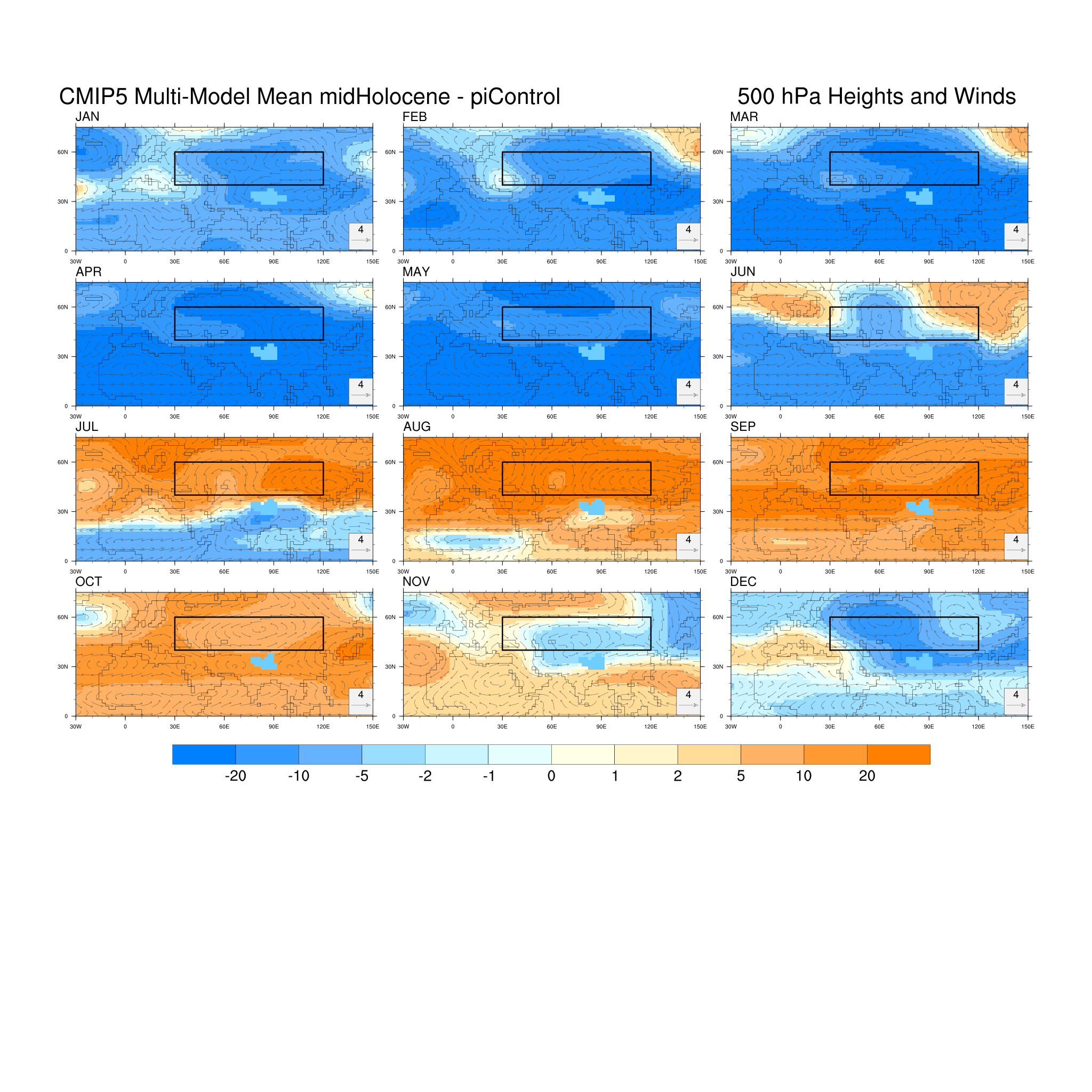


**Figure S20.** *midHolocene* minus *piControl* long-term mean differences in 500 hPa heights (zg) and winds (ua and va). Gray areas indicate regions where the land surface is above the 500 hPa level. The region of interest is shown by the black box.


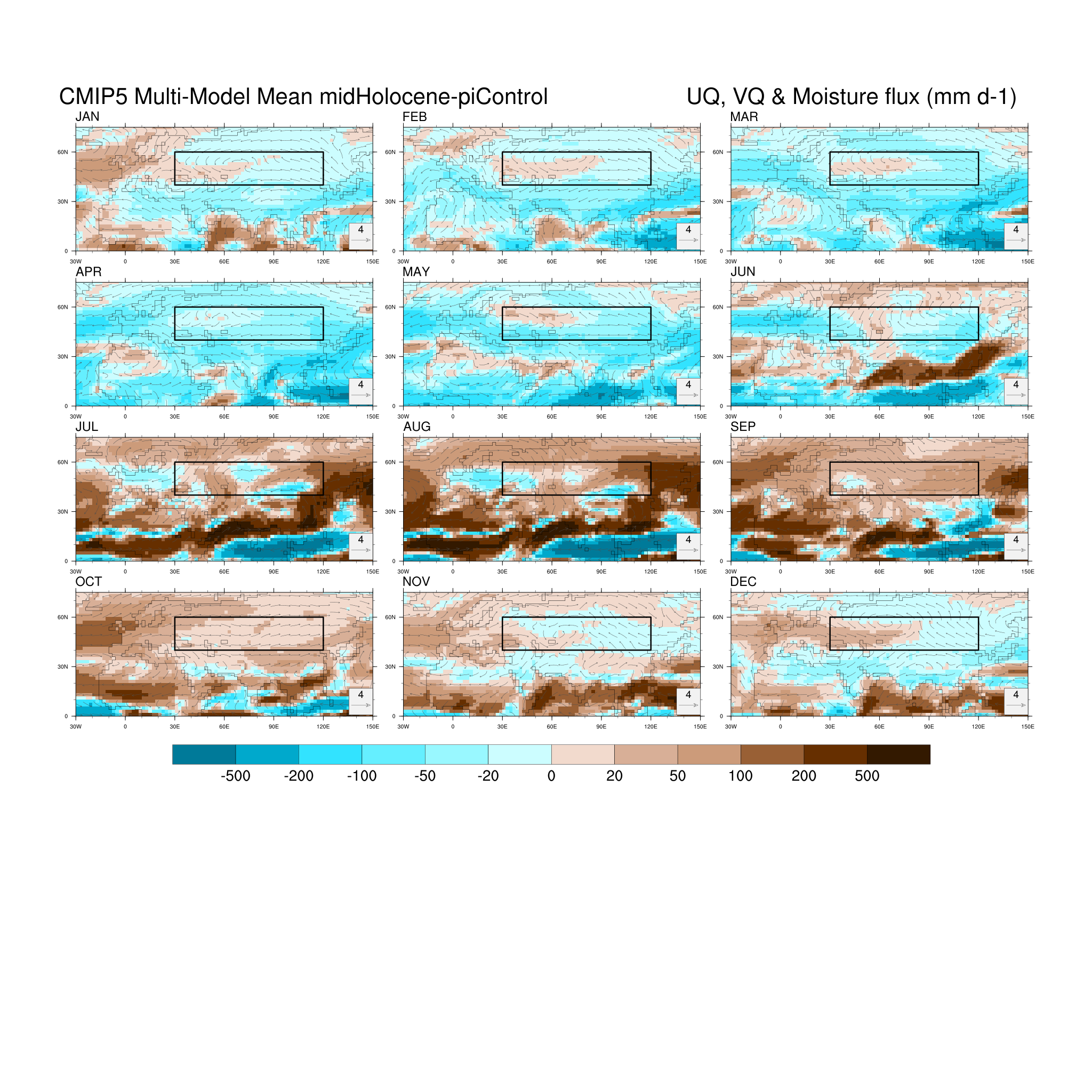


**Figure S21.** *midHolocene* minus *piControl* long-term mean differences of vertically integrate moisture flux (color) and its eastward (uq) and northward (vq) components (vector). The region of interest in this paper is shown by the black box.


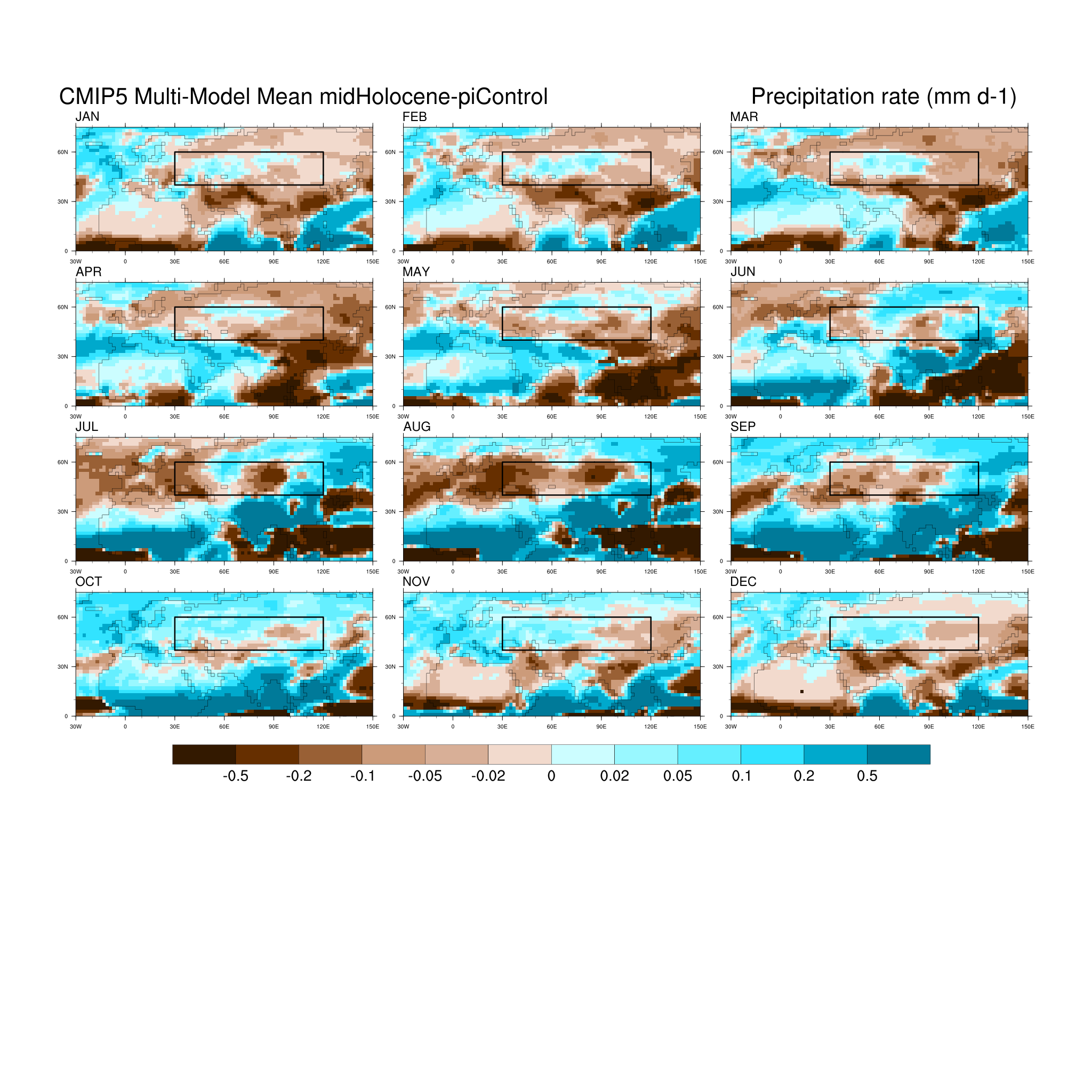


**Figure S22.** *midHolocene* minus *piControl* long-term mean differences in precipitation rate (pre). The region of interest is shown by the black box.


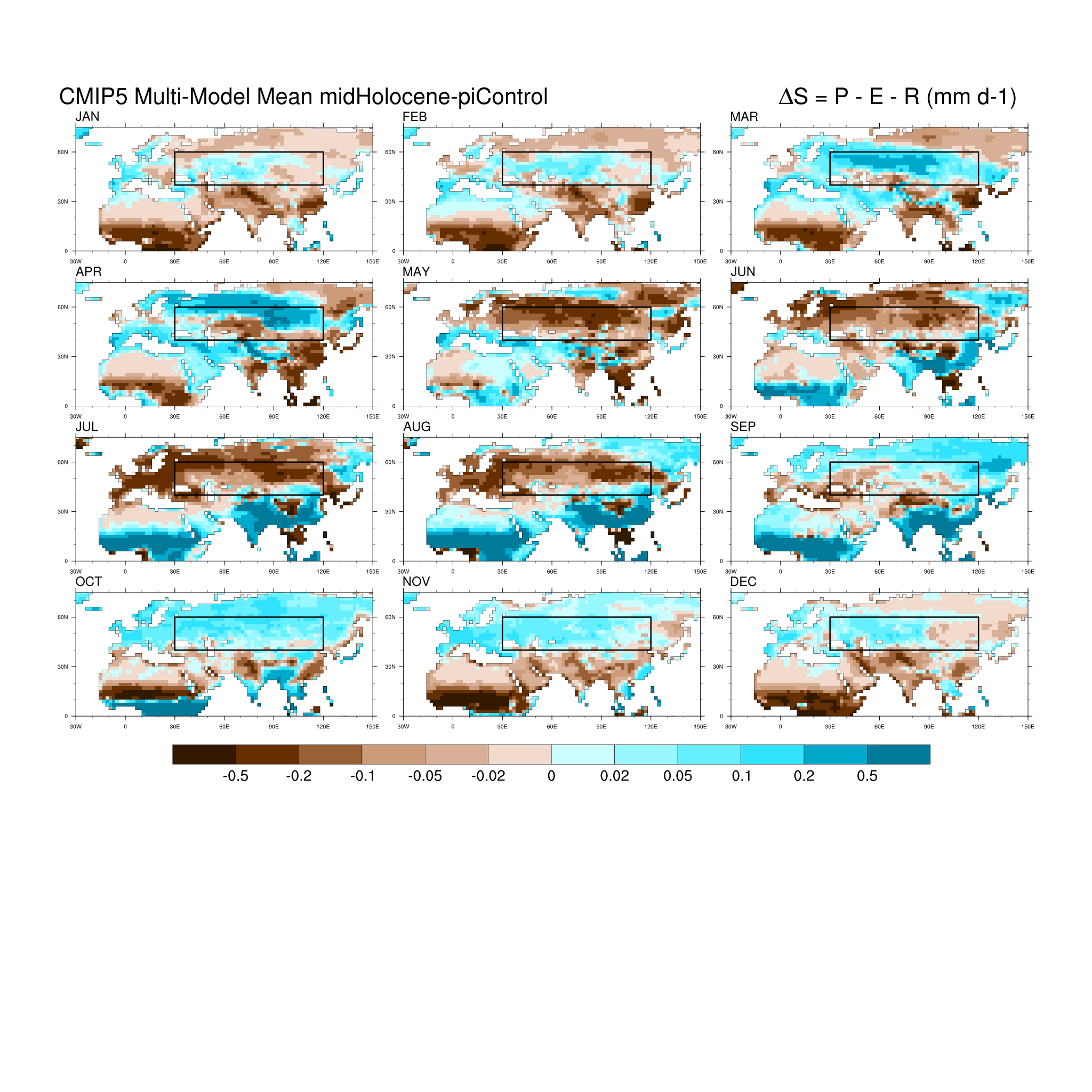


**Figure S23.** *midHolocene* minus *piControl* long-term mean differences in the change in soil moisture (dS). The region of interest in this paper is shown by the black box.


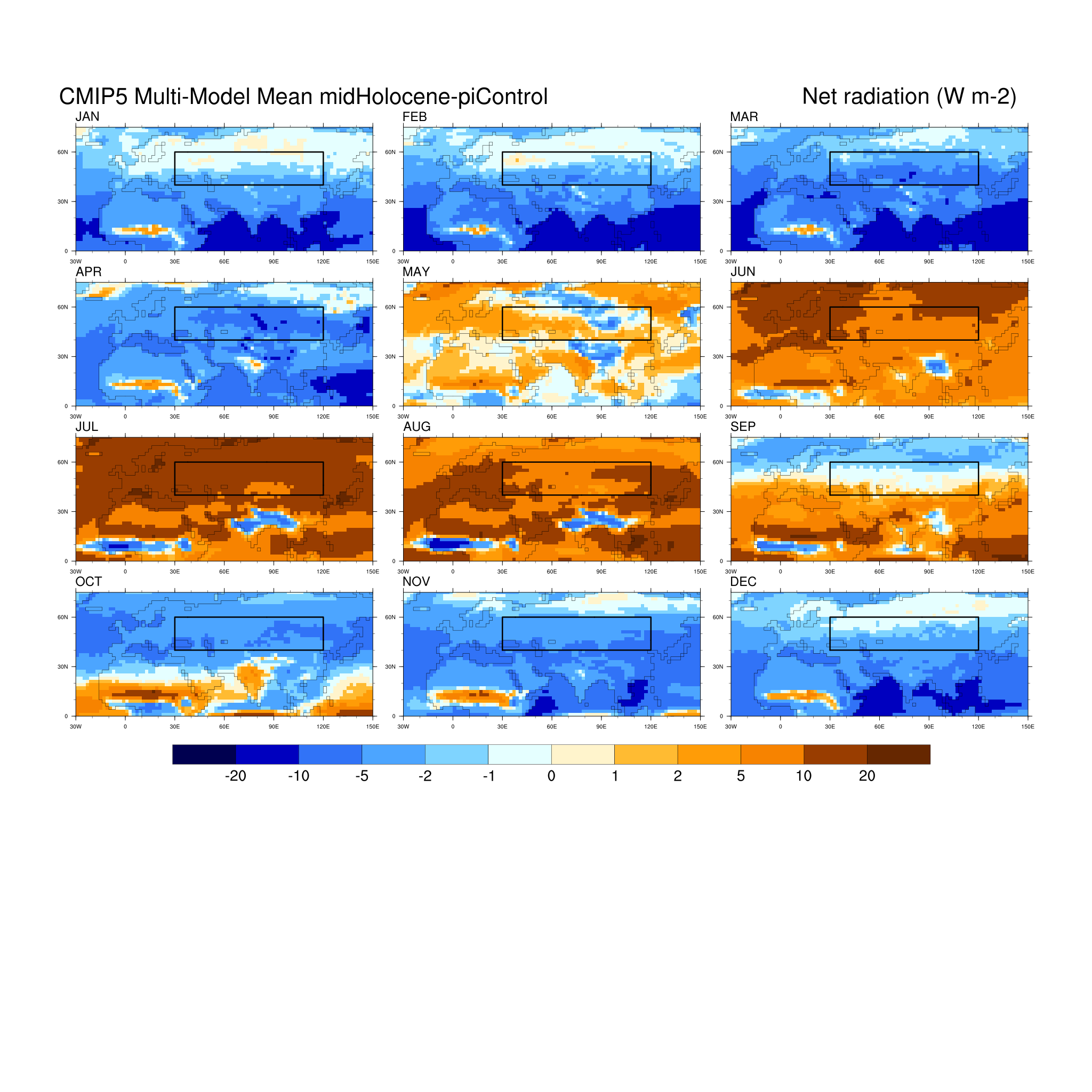


**Figure S24.** *midHolocene* minus *piControl* long-term mean differences in the change in net radiation (netrad). The region of interest is shown by the black box.


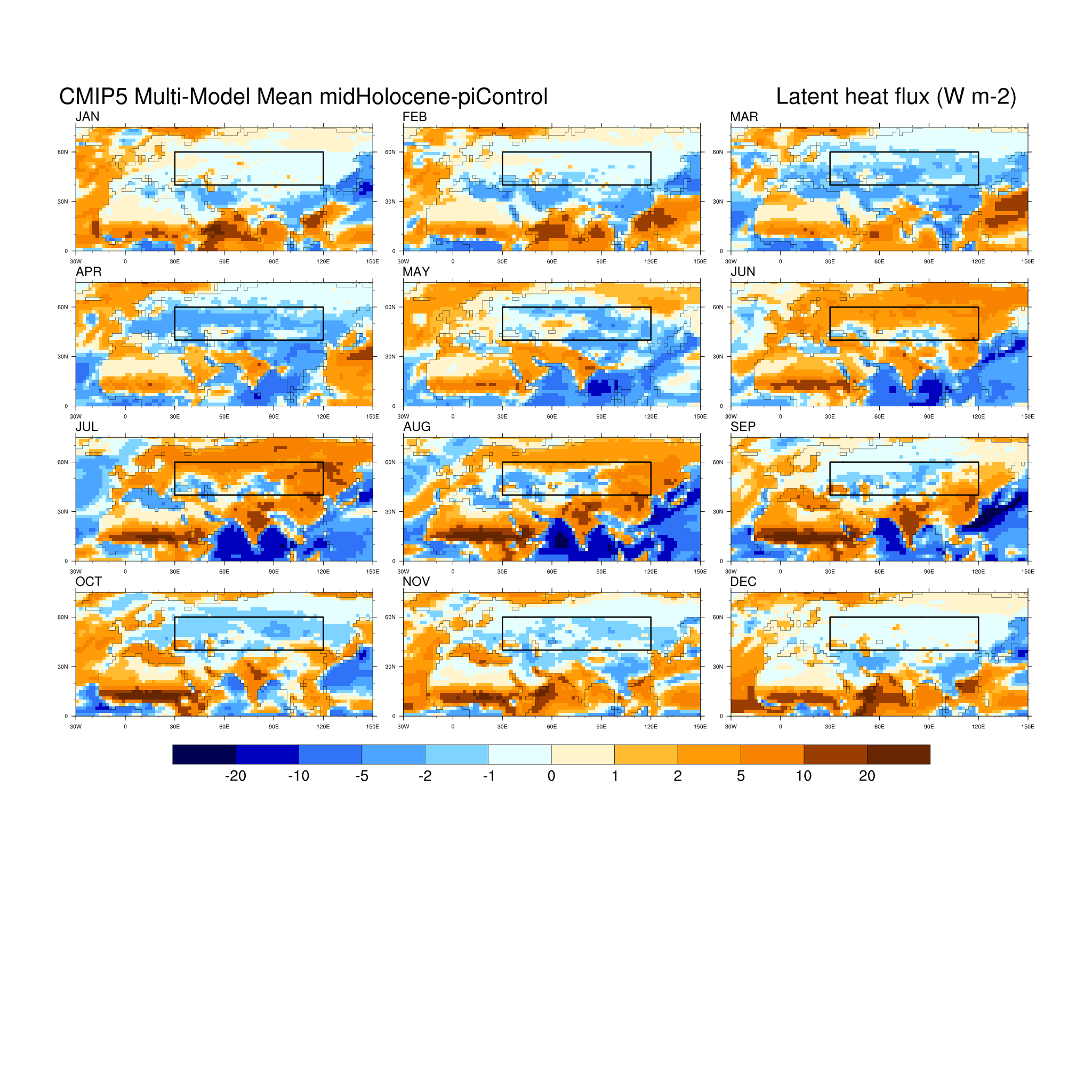


**Figure S25.** *midHolocene* minus *piControl* long-term mean differences in the change in latent heat flux (hfls), from the surface to the atmosphere (orange) or from the atmosphere toward the surface (blue). The region of interest in this paper is shown by the black box.


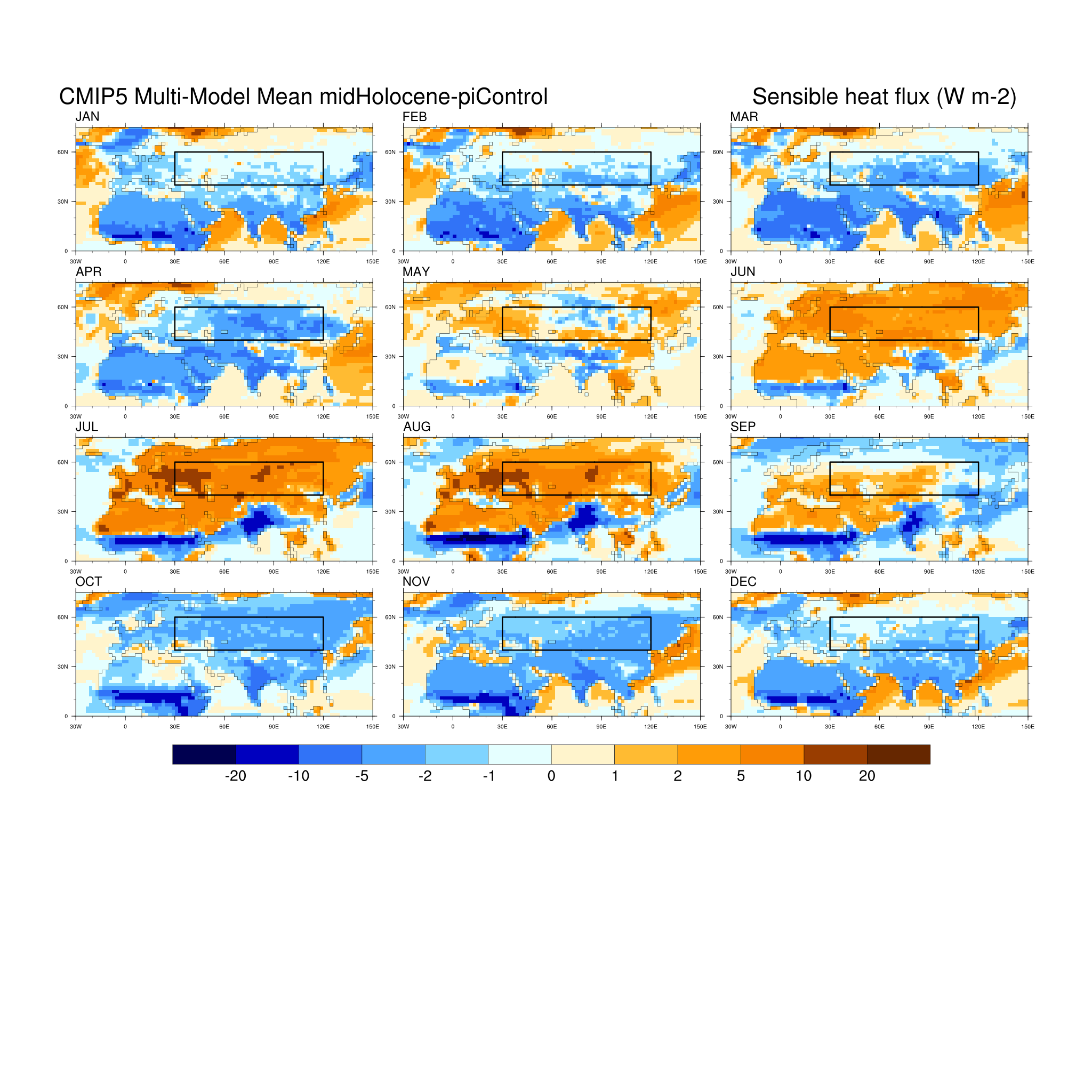


**Figure S26.** *midHolocene* minus *piControl* long-term mean differences in the change in sensible heat flux (hfss), from the surface to the atmosphere (orange) or from the atmosphere toward the surface (blue). The region of interest is shown by the black box.


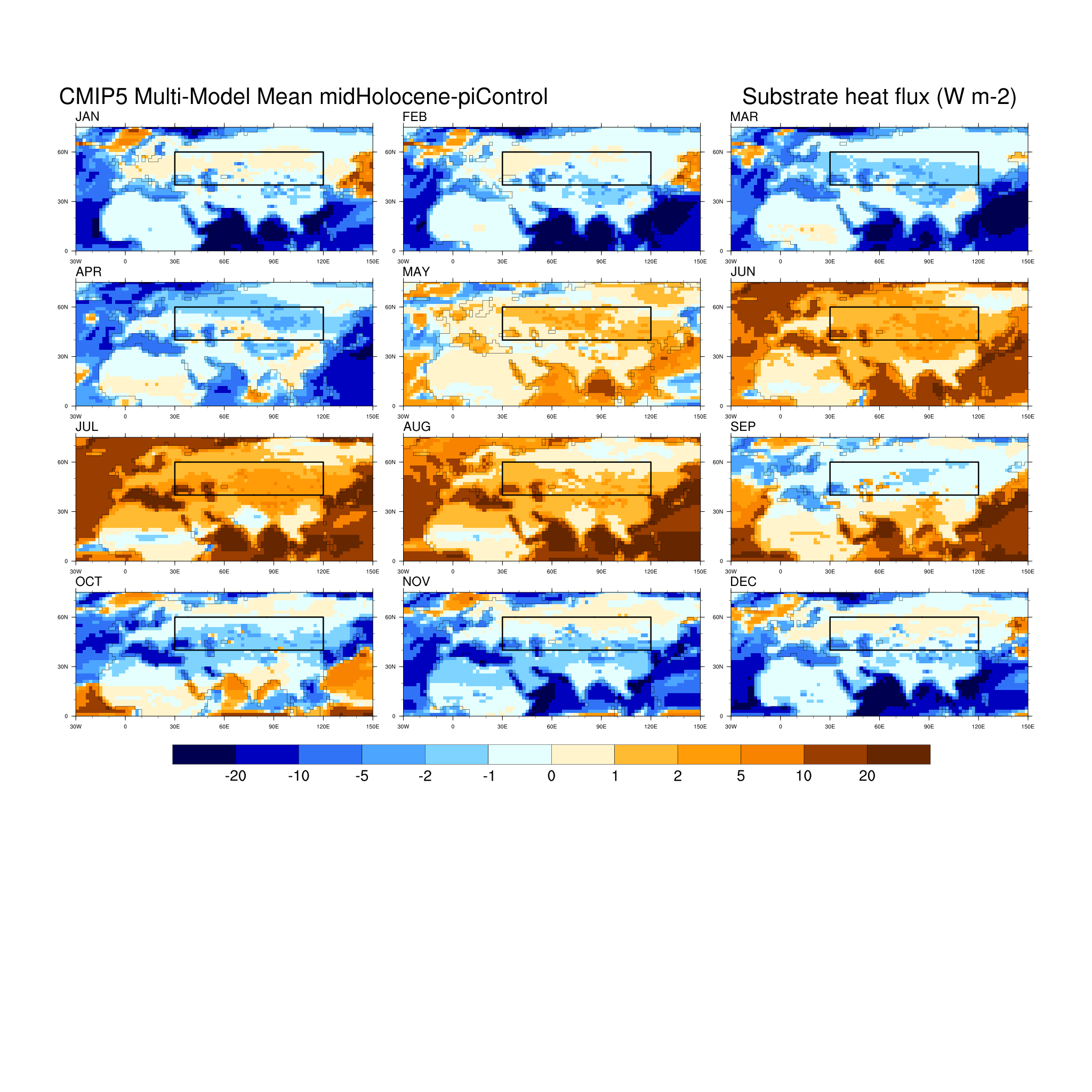


**Figure S27.** *midHolocene* minus *piControl* long-term mean differences in the change in the heat flux into (orange) or out of (blue) the substrate (hfsub). The region of interest in this paper is shown by the black box.


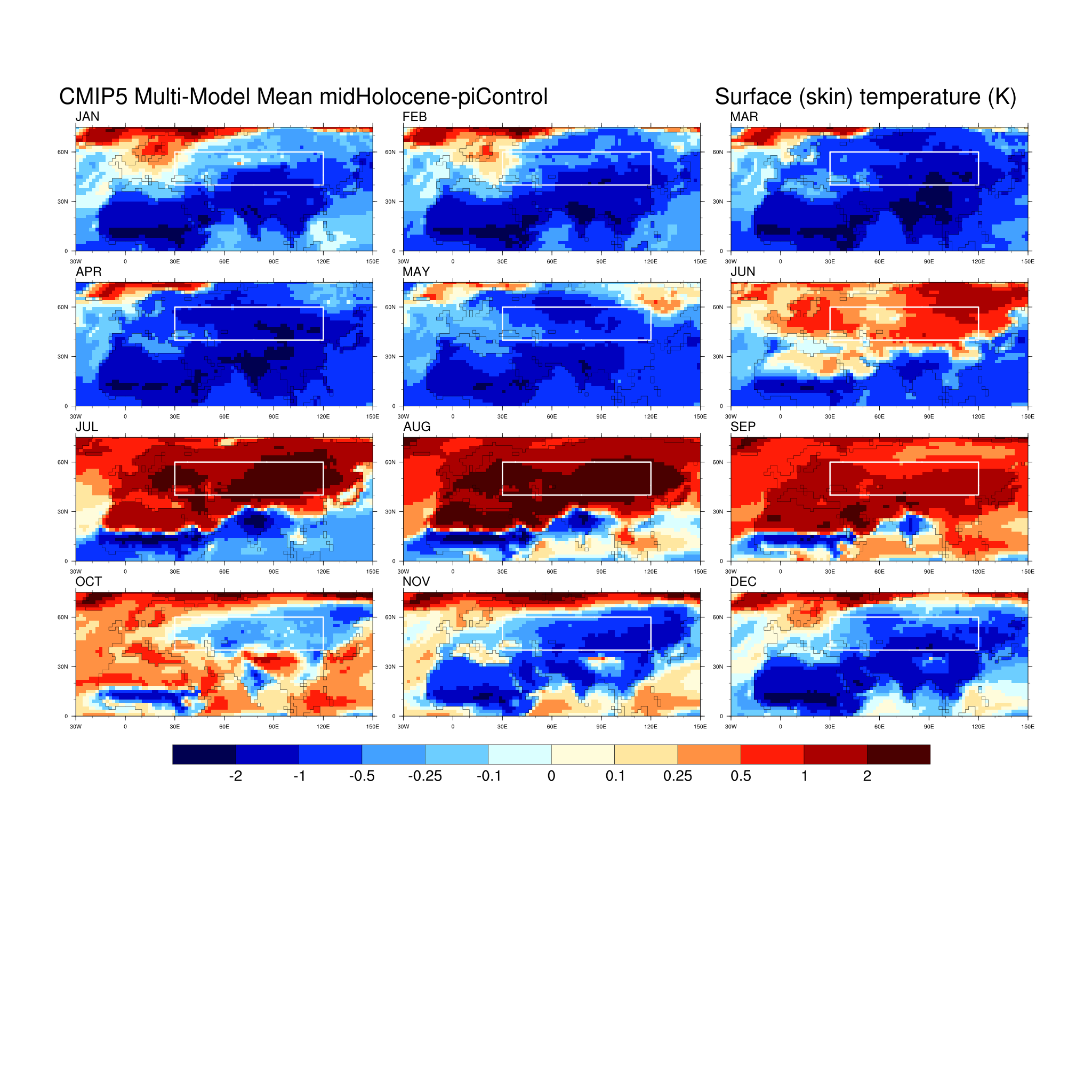


**Figure S29.** *midHolocene* minus *piControl* long-term mean differences in the change in surface (“skin”) temperature (ts). The region of interest is shown by the white box.


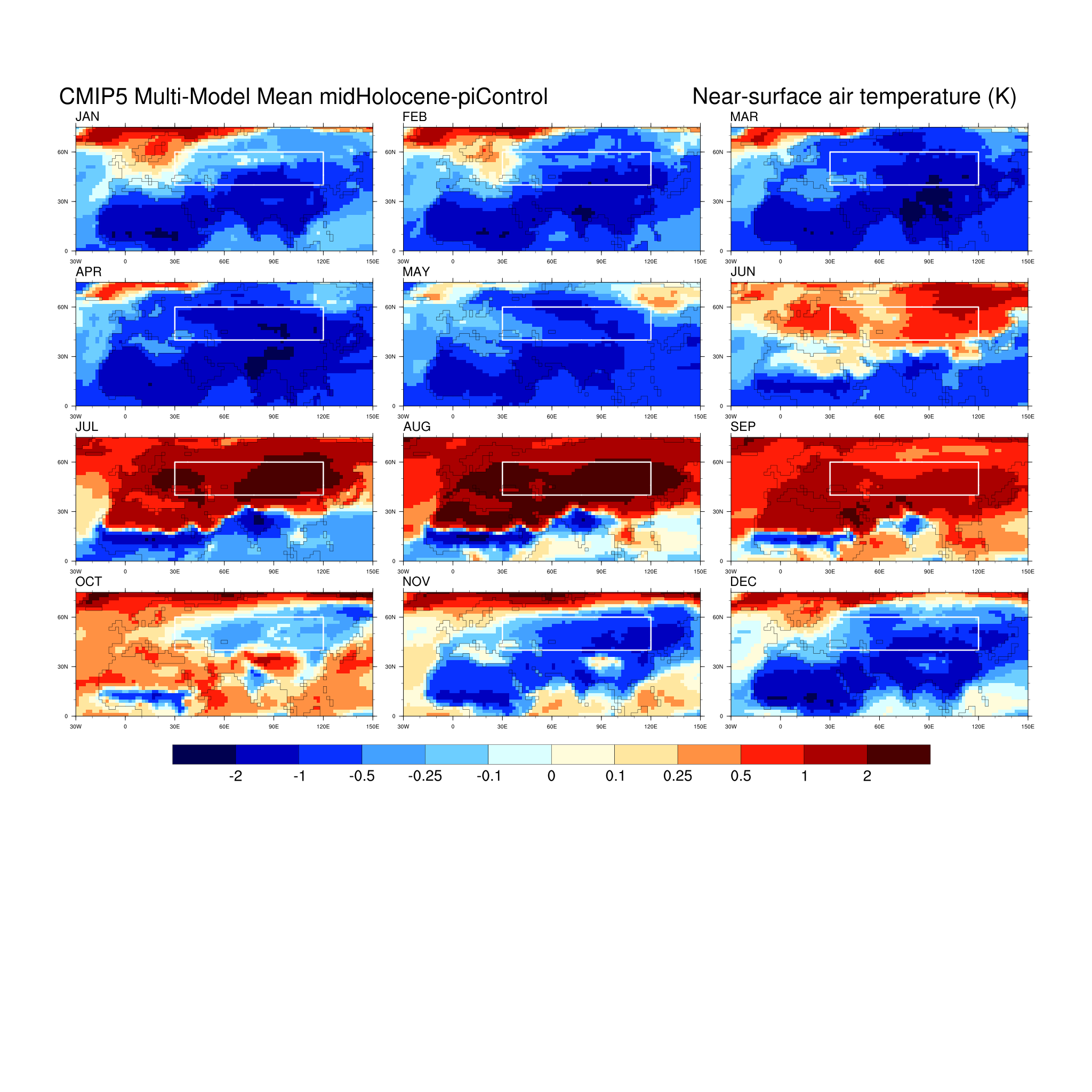


**Figure S29.** *midHolocene* minus *piControl* long-term mean differences in the change in near-surface temperature (tas). The region of interest in this paper is shown by the white box.


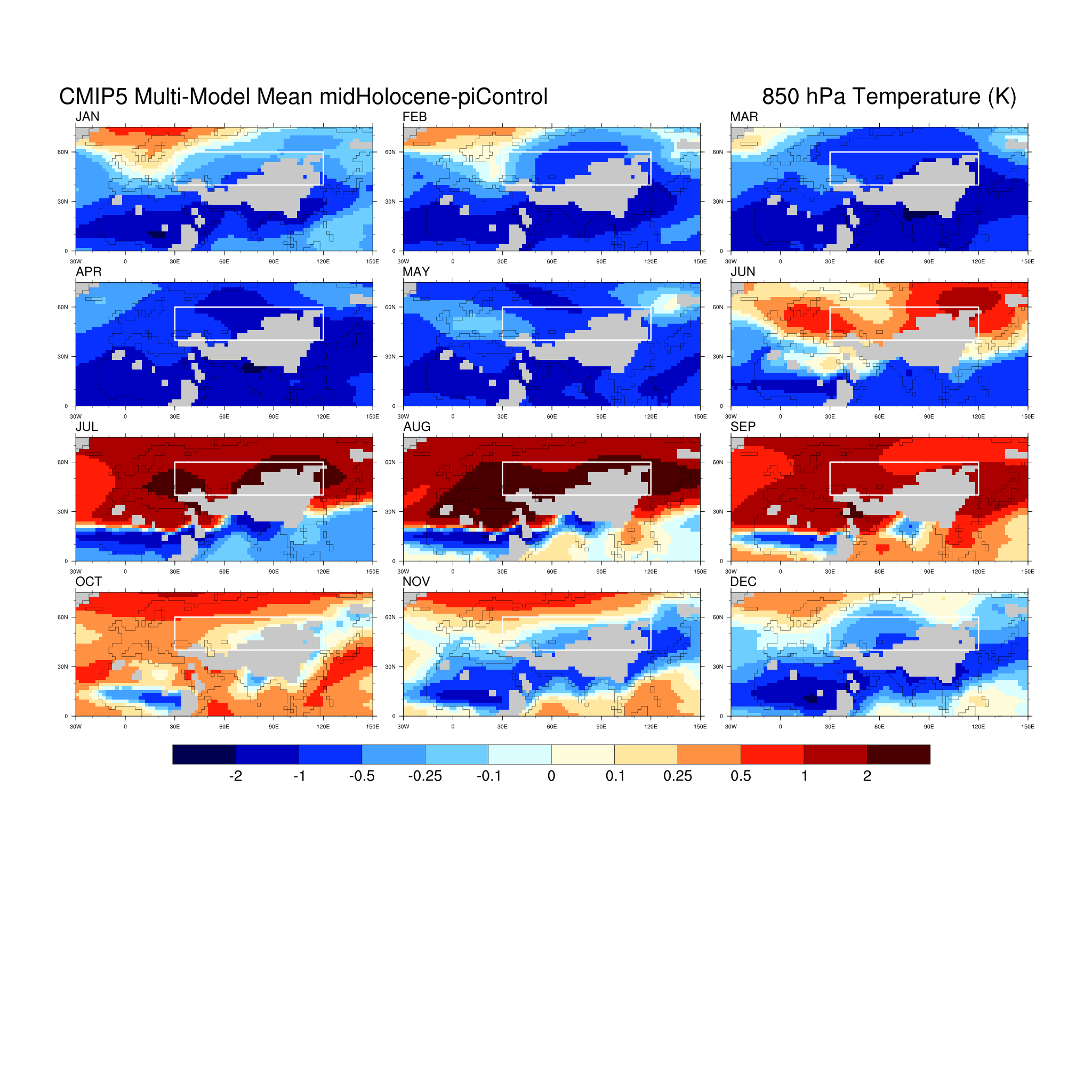


**Figure S30.** *midHolocene* minus *piControl* long-term mean differences in the change in 850 hPa temperature (ta850). Gray areas indicate regions where the land surface is above the 850 hPa level. The region of interest is shown by the white box.

Figure S31. Alternative version of Fig. 2, showing median values of *midHolocene* minus *piControl* differences (as black diamonds).

| **Table S1. Variables** | | |
| --- | --- | --- |
| **CMIP5 name** | **NCEP 2 name** | **CMIP5 long name** |
| tas | air.2m | near-surface air temperature |
| ts | skt | surface temperature |
| pr | prate | precipitation (rate) |
| hfls | lhtfl | surface upward latent heat flux |
| hfss | shtfl | surface upward latent heat flux |
| mrros | runof.sfc | surface runoff |
| mrso |  | total soil moisture |
| evspsbl |  | evaporation rate (NCEP 2 evspsbl – L_v_*lhtfl) |
| ta | air | air temperature (on standard pressure levels:) |
| ua | uwnd | eastward wind (on standard pressure levels:) |
| va | vwnd | northward wind (on standard pressure levels:) |
| zg | hgt | geopotential height (on standard pressure levels:) |
|  |  |  |
| **Derived or subsetted variables:** | | |
| Name |  | **Definition (in terms of CMIP5 variable names)** |
| dS |  | soil-moisture change (dS = pr – evap - mrros) |
| netrad |  | net radiation (netrad = rsds – rsus + lrds – rlus) |
| hfsub |  | substrate heat flux (hfsub = netrad – hfls – hfss) |
| pme |  | precipitation – evaporation (pme = pr – evap) |
| qmag |  | vertically integrated moisture flux (qmag = f(ua, va, hus, ps)) |
| uq |  | eastward vertically integrated moisture transport |
| vq |  | northward vertically integrated moisture transport |
| ta850 |  | 850 hPa temperature |
| hgt500 |  | 500 hPa geopotential height |
| tagrad |  | 500 hPa temperature gradient |
| zindex |  | 500 hPa zonal index |
|  |  |  |
| **Other variables used here:** | | |
| **CMIP5 name** | **NCEP name** | **CMIP5 long name** |
| psl | mslp | sea-level pressure |
| ps | pres.sfc | surface air pressure |
| hurs |  | near-surface relative humidity |
| uas | uwnd.10m | eastward near-surface wind |
| vas | vwnd.10m | northward near-surface wind |
| sfcWind | wspd.10, | near-surface wind speed |
| rlds | dswrf.sfc | surface downwelling longwave radiation |
| rlus | uswrf.sfc | surface upwelling longwave radiation |
| rsds | dlwrf.sfc | surface downwelling shortwave radiation |
| rsus | ulwrf.csv | surface upwelling shortwave radiation |
| clt | tcdc.eatm | total cloud fraction |
| snd | weasd.sfc | snow depth |
| wap | omega | omega (on standard pressure levels) |
| hus |  | specific humidity |
|  | rhum | relative humidity |

**Table S2. Models used in the analysis**

| **Archive** | **Model name** | **Type** | **Model components** |
| --- | --- | --- | --- |
| CMIP5 | BCC-CSM1-1 | OAC | BCC_AVIM1.0/MOM4/ SIS |
| CMIP5 | CCSM4 | OA | CAM4/POP2/CLM4/CICE4/CPL7 |
| CMIP5 | CNRM-CM5 | OA | ARPEGE-Climat V5.2.1, TL127L31/NEMO3.3.v10.6.6P/ORCA1degL42)/ GELATOV5.30/TRIPv1/SURFEXv5.1.c/OASIS 3 |
| CMIP5 | CSIRO-Mk3-6-0 | OA | AGCMv7.3.5/GFDL MOM 2.2 |
| CMIP5 | CSIRO-Mk3L-1-2 (UNSW) | OA | CSIRO Mk3/MOM2.2 |
| CMIP5 | FGOALS-s2 (LASG/IAP) | OA | SAMIL 2-4-7/LICOM/LASG ver.1 |
| CMIP5 | FGOALS-g2 (LASG/IAP) | OA | GMAIL/LICOM/CICE/CLM |
| CMIP5 | GISS-E2-R | OA | ModelE/Russell |
| CMIP5 | HadGEM2-CC | OAC | HadGAM2/HadGOM2/MOSES2/TRIFFID/UKCA/ diat-HadOCC |
| CMIP5 | HadGEM2-ES | OAC | HadGAM2/HadGOM2/MOSES2/TRIFFID/UKCA/ diat-HadOCC |
| CMIP5 | IPSL-CM5A-LR | OAC | LMDZ4_v5/ORCA2(NEMOV2_3)/ LIM2(NEMOV2_3) /PISCES/ORCHIDEEE |
| CMIP5 | MIROC-ESM | OAC | MIROC-AGCM (2010)/COCO3.4/SPRINTARS 5.00/NPZD/SEIB-DGVM |
| CMIP5 | MPI-ESM-P | OA | ECHAM6/MPIOM |
| CMIP5 | MRI-CGCM3 | OA | GSMUV/MRI.COM3/ HALv0.31 |

**Data Set S1.** Lake status at 6000 cal yr BP compared to present day.
