# Supplementary material for: Underlying causes of Eurasian midcontinental aridity in simulations of mid‐Holocene climate
Source: Geophys Res Lett. 2017 Sep 9;44(17):9020–8. doi: 10.1002/2017GL074476 (PMC5661745; doi:10.1002/2017GL074476)
Supplement: Supplementary file 2 — Data Set S1 [file GRL-44-9020-s002.pdf]

Data Set S1: Lake status at 6000 cal yr BP compared to present day.

Site Name	Latitude	Longitude	Elevation	6-0ka cal	Source or Reference
a'Mhuillinn Loch	55.7	-5.27	25	-1	GLSDB
Achit-Nur Lake	49.5	90.6	1435	-1	GLSDB
Ageröds mosse	55.93	13.43	55	-1	GLSDB
Ahung Co	32.0333	96.1	4575	1	Morrill et al. (2006)
Ahvenainen	61.03	25.11	122.2	0	GLSDB
Aiding Lake	42.67	89.27	-155	1	GLSDB
Antu Sinjärv Lake	59.13	26.33	94.6	1	GLSDB
Aral Sea	45	60	53	1	Boomer et al. (2000)
Argayesh Lake	55.58	60.89	250	0	GLSDB
Attersee	47.86	13.53	469	-1	GLSDB
Baijian Lake	39.15	104.17	1282	1	GLSDB
Baisuhai	42.59	115.93	2000	1	GLSDB
Balaton	46.8	17.5	105	-1	GLSDB
Balikun Lake	43.7	92.8	1575	1	GLSDB
Bangge Lake	31.75	89.57	4520	1	GLSDB
Bangong Co/Sumxi (Bangongcuo)	33.6667	79.42	4241	1	Gasse et al. (1996); Kong et al. (2007)
Bayan Nuur	50	94	932	1	Grunert et al. (2000)
Bayanchagan Lake	41.6	115.2	1355	1	Jiang and Liu (2007)
Bebrukas lake	54.12	24.55	160	1	GLSDB
Beilikekule Lake	36.72	89.05	4680	1	GLSDB
Beloe Mire	62.33	39	135	0	GLSDB
Beloe-Chernoe Lakes	55.72	37.35	145	1	GLSDB
Bezdonnoe Mire	62	32.5	123	0	GLSDB
Bielersee	47.1	7.1	429	-1	GLSDB
Black Loch	56.25	-3.17	90	-1	GLSDB
Boguda lake	63.67	123.25	117	0	GLSDB
Bol'shoi Kisegach Lake	55.04	60.38	317	-1	GLSDB
Borovoe Lake	53.08	70.27	321	1	GLSDB

Borralan Loch	58.05	-4.9	139	0	GLSDB
Bosten Lake	42.0833	87.05	1048	1	Wünnemann et al. (2003); Wünnemann et al. (2006); Huang et al. (2009)
Bysjön	55.67	13.53	22	-1	GLSDB
Cam	58.08	-5	123.5	0	GLSDB
Castiglione	41.89	12.76	44	-1	GLSDB
Cepkeliu Raistas Marsh	54.02	24.52	130.5	-1	GLSDB
Chabada Lake	61.98	129.37	245	0	GLSDB
Chaerhan Salt Lake	36.93	94.99	2675	1	GLSDB
Chagannur	43.27	112.9	920	-1	GLSDB
Chaiwopu Lake	43.5	87.9	1092	-1	GLSDB
Chalain	46.68	5.77	488	-1	GLSDB
Chany Lake	54.82	77.5	106.1	0	GLSDB
Chechkino Mire	62.25	34	55	1	GLSDB
Chervonoe Lake	52.38	28	136	0	GLSDB
Chistoe Lake	55.78	38.32	160	1	GLSDB
Clairvaux	46.57	5.75	526	-1	GLSDB
Cregganmore	54.25	-9.6	60	1	GLSDB
Crose Mere	52.83	-2.83	87	-1	GLSDB
Daba-Nur Lake	48.2	98.8	2465	1	GLSDB
Dachaidan-Xiaochaidan Salt Lakes	37.5	95.37	3110	1	GLSDB
Dali Lake, Hunshandake Sandy Lands	43.25	116.5833	1350	1	Xiao et al. (2008); Yang et al. (2015)
Diss Mere	52.37	1.1	26	-1	GLSDB
Dlinnoe Mire	62.32	33.85	66	1	GLSDB
Dolgoe Lake	56.07	37.32	200	-1	GLSDB
Dood-Nur Lake	51.33	99.38	1538	1	GLSDB
Dubh Lochan	56.15	-4.6	75	1	GLSDB
Eastern Juyan palaeolake	41.89	101.85	892	1	Herzschuh et al. (2004)
Ellasjøen	74.38	19.01	20.8	1	GLSDB
Endletvatn	69.73	19.08	35	0	GLSDB

Erhai	25.84	99.98	1973	1	Shen et al. (2005)
Erjichuoer	45.23	116.5	829.2	0	GLSDB
Federsee	48.17	9.58	578	-1	GLSDB
Fucino	42	13.55	667	-1	GLSDB
Galichskoe Lake	58.4	42.28	101.2	-1	GLSDB
Garten	57.22	-3.7	220	0	GLSDB
Glubelka lake	54.95	26.42	165.8	0	GLSDB
Goplo	52.6	18.31	77	0	GLSDB
Gotnavolok Mire	62.17	33.75	88	1	GLSDB
Gounongcuo	34.35	92.2	4670	1	GLSDB
Großer Plöner See	54.17	10.42	40	-1	GLSDB
Gun-Nur Lake	50.25	106.6	600	1	GLSDB
Hafratjörn	65.83	-20.1	97	1	GLSDB
Hakojärvi	61.25	25.2	145.6	1	GLSDB
Hobschensee	46.25	8.01	2017	0	GLSDB
Hockham Mere	52.5	0.83	33	1	GLSDB
Hoton-Nur Lake	48.67	88.3	2083	1	GLSDB
Hovsgol Nuur	51	101.2	1645	-1	Prokopenko et al. (2007)
Hubsugul Lake	50.53	100.17	1645	-1	GLSDB
Huguangyan	21.15	110.2833	87	1	Mingram et al. (2004); Sheng et al. (2017)
Hulun Nuur (Hulun Lake)	48.93	117.23	545	1	Wang and Ji (1995)
Hurleg Lake, Qaidam	37.2833	96.9	2817	-1	Zhao et al. (2007): Zhao et al. (2010); Zhao et al. (2013)
Il'men Lake	58.3	31.23	18	0	GLSDB
Ioannina	39.66	20.88	469	1	GLSDB
Isohattu	68.6	23.6	386	-1	GLSDB
Issyk-kul Lake	42.5	77.1	1606	1	Ricketts et al. (2001); Ferronskii et al. (2003)
Jierstivaara	68.6	23.63	456	-1	GLSDB
Jilantai	39.75	105.7	1023	1	GLSDB
Juokojauratj	66.6	19.27	400	-1	GLSDB
Kalina Mire	59.37	27.42	70	0	GLSDB

Kanent'yavr Lake	68.8	34.3	182.9	1	GLSDB
Karakul	39.0176	73.5327	NA	1	Heinecke et al. (2016)
Karas'e Lake	53.03	70.22	435	-1	GLSDB
Kastoria	40.55	21.32	650	1	GLSDB
Kepa	49.4	22	320	0	GLSDB
Khimaditis	40.6	21.57	560	1	GLSDB
Khomustakh Lake	63.82	121.62	120	1	GLSDB
Kirikumäe Lake	57.67	27.25	183	1	GLSDB
Kissalammi	61.28	24.35	90.4	1	GLSDB
Kolno	53.77	23.01	121	0	GLSDB
Kotokol Lake	52.83	108.17	460	0	GLSDB
Kovdor Lake	67.65	30.88	160	1	GLSDB
Krageholmssjön	55.5	13.73	43	-1	GLSDB
Krasnokamsk Mire	58.05	55.7	90	1	GLSDB
Krivoe Lake	55.14	29.07	131	1	GLSDB
Kroktjärnen	66.23	20.85	58	0	GLSDB
Kruklin	54.07	21.87	127	1	GLSDB
Kumphawapi, Thailand	17.1833	103.0333	170	1	Chawchai et al. (2013)
Kyrösjärvi	61.75	23.17	83	-1	GLSDB
Lac d'Issarlès	44.8	4.07	997	0	GLSDB
Lacha Lake	62.59	37.67	118	1	GLSDB
Lago di Ganna	45.87	8.83	452	0	GLSDB
Lagoa Comprida	39.75	-8	1600	1	GLSDB
Lake Xingyun	24.3368	102.7817	1723	-1	Zhou et al. (2015); Hodell et al. (1999)
Laksa Mire	62.8	40.58	109	1	GLSDB
Lampellonjärvi-Lamminjärvi	62.07	29.07	108.6	1	GLSDB
Landos	44.83	3.8	1000	0	GLSDB
Le Grand Lemps	45.47	5.42	456	1	GLSDB
Léman	46.91	7.25	373	0	GLSDB
Lerstadvatn	62.5	6.5	44	0	GLSDB
Lilla Gloppsjön	59.81	14.62	198	-1	GLSDB

Linggo Co	33.9	89	5059	1	Pan et al. (2012)
Linton Loch	55.87	-2.63	91.5	-1	GLSDB
Ljustjärnen	59.76	14.48	183	-1	GLSDB
Llyn Clyd	53.08	-4.17	746.8	-1	GLSDB
Lobsigensee	47.03	7.3	514.4	-1	GLSDB
Lomatjörn	64.33	-20.33	100	1	GLSDB
Longquanhu	30.87	112.03	150	1	GLSDB
Lop Basin	40.29	90.8	787	1	GLSDB
Lovojärvi	61.08	25.03	108.2	0	GLSDB
Lubanas Lake	56.77	26.92	90.8	0	GLSDB
Lukcze	51.5	23	170	0	GLSDB
Madjagara Lake	64.83	120.97	160	0	GLSDB
Malo Jezero	42.78	17.35	0	0	GLSDB
Manasi Lake	45.45	86	251	1	GLSDB
Maybalyc Lake	53.15	70.13	306	0	GLSDB
Megalake Tengger	39	104	1345	1	Zhang et al. (2004)
Melekhovo Mire	56.83	38.5	150	0	GLSDB
Melynllyn	53.12	-4.12	632.5	0	GLSDB
Michurinskoe Lake	60.52	29.98	94	1	GLSDB
Mitrofanovskoe Lake	67.83	59	116.8	0	GLSDB
Mokhovoe Lake	53.77	64.25	178	1	GLSDB
Mondsee	47.83	13.37	475	1	GLSDB
Moshkarnoe Mire	62.25	34.25	58	1	GLSDB
Mutnoe Lake	55.5	31.78	180	-1	GLSDB
Nancun	24.75	110.42	160	-1	GLSDB
Naroch Lake	54.85	26.85	120	-1	GLSDB
Nero Lake	57.17	39.48	93	-1	GLSDB
Nerskoe Lake	56.08	37.39	165	-1	GLSDB
Ningjingbo	37.25	114.96	26	1	GLSDB
Nong Han Kumphawapi, Thailand	17.1333	103.0167	NA	0	Penny et al. (1996)
Nosue Mire	64.58	30.58	165	0	GLSDB

Nuochaga Lake	61.3	129.55	250	0	GLSDB
Nussbaumersee	47.65	8.45	434	-1	GLSDB
Old Buckenham Mere	52.5	1.02	35	0	GLSDB
Oltush Lake	51.7	23.96	158.3	0	GLSDB
Osintsevo Mire	57.37	57.63	148	1	GLSDB
Paanayarvi Lake	66.27	29.95	136.6	-1	GLSDB
Päidre Lake	58.27	25.5	50.6	-1	GLSDB
Palu	45.01	13.7	0	1	GLSDB
Paru Co	29.796	92.352	4845	1	Bird et al. (2014)
Pashennoe Lake	49.37	75.4	871	-1	GLSDB
Pasmlambina Lake	66.7	35.45	122	1	GLSDB
Pelléautier	44.52	6.18	975	-1	GLSDB
Peschanoe Lake	51.98	25.48	139.3	1	GLSDB
Pieni Majaslampi	60.31	24.58	97.3	-1	GLSDB
Pityoulish	57.2	-3.8	210	-1	GLSDB
Pluvis	45.63	5.63	215	0	GLSDB
Pølsa	74.5	18.93	14	1	GLSDB
Qinghai	36.8917	100.1917	3193	0	Madsen et al. (2008); Wang et al. (2015)
Raigastvere Lake	58.6	26.73	51.8	-1	GLSDB
Rangkul-Shorkul Lakes	38.52	74.2	3779	1	GLSDB
Ranviken	56.27	14.3	81	-1	GLSDB
Razlomnoe Mire	62.24	34.53	53	1	GLSDB
Richi Lake	55.68	26.72	146.2	1	GLSDB
Rotsee	47.15	8.31	419	0	GLSDB
Rousses	46.8	5.2	0	1	GLSDB
Rudushskoe Lake	56.5	27.55	150	1	GLSDB
Rugozero Mire	64.08	32.52	130	0	GLSDB
Sala Us/Dagouwan	38	108	NA	1	Li et al. (2012)
Salawusu Palaeolake	37.7	108.6	1300	1	GLSDB
Sandsjön	56.75	13.42	157.8	0	GLSDB
Sanguijuelas	42.1	-6.73	1050	1	GLSDB

Sanjiang Plain	47.58493	133.5001	71	1	Zhang et al. (2014)
Sarkkilanjärvi	61.75	23.1	87	0	GLSDB
Saviku Mire	58.42	27.24	31	0	GLSDB
Schleinsee	47.75	9.5	474	1	GLSDB
Schukozero Lake	62.35	39.83	165	-1	GLSDB
Sea Mere	52.57	1	38	-1	GLSDB
Seeburger See	51.5	10.17	156	-1	GLSDB
Seling Co	31.75	88.9334	4530	1	Morinaga et al. (1993); Gasse et al. (1996)
Shayema Lake	28.83	102.2	2400	-1	GLSDB
Sheeauns Lough	53.55	-10.05	18	0	GLSDB
Shventoji Mire	55	21.25	5	1	GLSDB
Sindorskoe Lake	60.77	52	130	-1	GLSDB
Skinkevatna	74.48	18.81	19.3	1	GLSDB
Somino Lake	56.6	38.8	134.4	1	GLSDB
Sonkul/Sonkel	41.8097	75.1272	3016	1	Mathis et al. (2012); Huang et al. (2014)
Sporovskoe Lake	52.33	25.33	142	-1	GLSDB
Steklin	52.95	19	73.3	0	GLSDB
Sudoble Lake	54.03	28.1	165	1	GLSDB
Tangra Yum Co	31.1	86.63	NA	1	Long et al. (2012); Rades et al. (2015)
Tegeler See	52.58	13.25	35	1	GLSDB
Telmen Lake	48.8	97.3	1789	-1	Peck et al. (2002); Fowell et al. (2003)
Terkhiin-Tsagan-Nur	48.15	99.7	2060	-1	GLSDB
Tesovo-Netyl'skoe Mire	58.92	30.9	58.5	0	GLSDB
Torreberga	55.37	13.14	8	0	GLSDB
Trostenskoe Lake	55.86	36.48	195	1	GLSDB
Trummen	56.87	14.75	161	0	GLSDB
Tur Lake	51.67	24.3	155	1	GLSDB
Tuuljärve Lake	57.69	27.14	257	1	GLSDB
Työtjärvi	60.98	25.47	142.8	1	GLSDB
Ugii Nuur	47.7333	102.7667	1328	0	Schwanghart et al. (2008)
Uvil'dy Lake	55.53	62.37	273.4	-1	GLSDB

Vaharu Mire	59.34	24.37	45	0	GLSDB
Valdaiskoe Lake	57.98	33.27	192.5	-1	GLSDB
Vanhalampi	66.37	29.58	205	-1	GLSDB
Växjösjön	56.87	14.75	161	-1	GLSDB
Vegoritis	40.75	21.75	570	1	GLSDB
Vielången	56.19	13.17	93	-1	GLSDB
Vishnevskoe Lake	60.5	29.52	15	1	GLSDB
Vorkalu Lake	56.33	27.5	154.3	-1	GLSDB
Vozhe Lake	60.43	39.08	121	-1	GLSDB
Wielkie Gacno	53.73	17.2	130	0	GLSDB
Wulanwula Lake	34.8	90.5	4854	1	GLSDB
Wulun Lake	47.2	87.3	478	1	Jiang et al. (2007)
Xiaoshazi Lake	36.97	90.73	4106	1	GLSDB
Xidadianzi	42.33	126.37	614	0	GLSDB
Xingkai Lake (Khanka Lake)	45.17	132.17	69	0	GLSDB
Xinias	39.07	22.26	500	1	GLSDB
Yanhaizi Lake	40.2	108.4	1180	0	Chen et al. (2003)
Zabuye Lake	31.35	84.07	4421	1	GLSDB
Zaozer'e Lake	55.02	31.92	175	-1	GLSDB
Zhacang Caka	32.6	82.38	4328	1	GLSDB
Zhuye Lake	39.15	103.7	1290	1	Zhang et al. (2004); Li et al. (2014a, b)

Notes

6-0ka cal: provides information on lake status at 6000 cal yr BP compared to present day, 0 cal yr BP, where positive values mean the lake was higher at 6000 cal yr BP than it is today, negative values mean the lake was lower at 6000 cal yr BP than it is today, and 0 indicates no change in lake status between the two time periods.

GLSDB: indicates that the lake status record has been taken from the Global Lake Status Database (http://www.bridge.bris.ac.uk/projects/GLSDB). The changes in lake status are derived from the continuous reconstructions provided for each individual lake, where the dates of the changes between status classes have been converted from the original radiocarbon age to calendar years, using the OxCal program (https://c14.arch.ox.ac.uk/oxcal.html) .

References

Bird, B.W., Polisar, P.J., Lei, Y., Thompson, L.G., Yao, T., Finney, B.P., ... Steinman, B.A. (2014), A Tibetan lake sediment record of Holocene Indian summer monsoon variability, *Earth and Planetary Science Letters*, *399*, 92-102. doi:10.1016/j.epsl.2014.05.017

Boomer, I., Aladin, N., Plotnikov, I., & Whatley, R. (2000), The palaeolimnology of the Aral Sea: a review, *Quaternary Science Reviews*, *19*(13), 1259-1278. doi:10.1016/S0277-3791(00)00002-0

Chawchai, S., Chabangborn A., Kylander, M. Löwemark, L., Mörth, M., Blaaw, M., … Wolfarth, B. (2013) Lake Kumphawapi — An archive of Holocene paleoenvironmental and paleoclimatic changes in northeast Thailand. *Quat. Sci. Rev.* 68, 59-75, doi:10.1016/j.quascirev.2013.01.030

Chen, F. H., Wu, W. Holmes, J.A. Madsen, D.B., Zhu, Y. Jin, M., & Oviatt, C.B. (2003), A mid-Holocene drought interval as evidenced by lake desiccation in the Alashan Plateau, Inner Mongolia, China, *Chin. Sci. Bull.*, 48, 1401–1410.

Ferronskii, V. I., V. A. Polyakov, V. S. Brezgunov, L. S. Vlasova, Y. A. Karpychev, A. F. Bobkov, … K. Rasmussen (2003), Variations in the Hydrological Regime of Kara-Bogaz-Gol Gulf, Lake Issyk-Kul, and the Aral Sea Assessed Based on Data of Bottom Sediment Studies, *Water Resources*, 30(3), 252-259, doi:10.1023/a:1023826011601.

Fowell, S. J., Hansen, B. C. S., Peck, J. A., Khosbayar, P., & E. Ganbold (2003), Mid to late holocene climate evolution of the lake telmen basin, north central mongolia, based on palynological data, *Quaternary Research*, 59(3), 353-363, doi:10.1016/S0033-5894(02)00020-0.

Gasse, F., Fontes, J.C., Van Campo, E., & Wei, K. (1996), Holocene environmental changes in Bangong Co basin (Western Tibet). Part 4: Discussion and conclusions, *Palaeogeography, Palaeoclimatology, Palaeoecology*, *120*(1), 79-92. doi:10.1016/0031-0182(95)00035-6

Grunert, J., Lehmkuhl, F., & Walther, M. (2000), Paleoclimatic evolution of the Uvs Nuur basin and adjacent areas (Western Mongolia), *Quaternary International*, *65*, 171-192. doi:10.1016/S1040-6182(99)00043-9

Kong, P., Na, C., Fink, D., Huang, F., & Ding, L. (2007), Cosmogenic 10Be inferred lake-level changes in Sumxi Co basin, Western Tibet, *Journal of Asian Earth Sciences*, *29*(5), 698-703. doi:10.1016/j.jseaes.2006.04.010

Heinecke, L., Mischke, S., Adler, K., Barth, A., Biskaborn, B. K., Plessen, B., … Herzschuh, U. (2016), Late Pleistocene to Holocene climate and limnological changes at Lake Karakul (Pamir Mountains, Tajikistan), *Clim. Past Discuss.*, *2016*, 1-30, doi:10.5194/cp-2016-34.

Herzschuh, U., Tarasov, P., Wünnemann, B., & Hartmann, K. (2004), Holocene vegetation and climate of the Alashan Plateau, NW China, reconstructed from pollen data, *Palaeogeography, Palaeoclimatology, Palaeoecology*, *211*(1), 1-17. doi:10.1016/j.palaeo.2004.04.001

Hodell, D.A., Brenner, M., Kanfoush, S.L., Curtis, J.H., Stoner, J.S., Xueliang, S., ... Whitmore, T.J. (1999), Paleoclimate of Southwestern China for the Past 50,000 yr Inferred from Lake Sediment Records, *Quaternary Research*, *52*(3), 369-380. doi:10.1006/qres.1999.2072

Huang, X., Oberhänsli, H., von Suchodoletz, H., Prasad, S., Sorrel, P., Plessen, B., ... Usubaliev, R. (2014), Hydrological changes in western Central Asia (Kyrgyzstan) during the Holocene as inferred from a palaeolimnological study in lake Son Kul, *Quaternary Science Reviews*, *103*, 134-152. doi:10.1016/j.quascirev.2014.09.012

Huang, X.Z., Chen, F.H., Fan, Y.X., & Yang, M.L. (2009), Dry late-glacial and early Holocene climate in arid central Asia indicated by lithological and palynological evidence from Bosten Lake, China, *Quaternary International*, *194*(1), 19-27. doi:10.1016/j.quaint.2007.10.002

Jiang, Q. F., Shen, J., Liu, X.Q., Zhang, E.L., & Xiao, X.Y. (2007), A high resolution climatic change since Holocene inferred from multi-proxy of lake sediment in westerly area of China, *China Sci. Bull.*, 52, 1970–1979.

Jiang, W.Y., & Liu, T.S. (2007), Timing and spatial distribution of mid-Holocene drying over northern China: Response to a southeastward retreat of the East Asian Monsoon, *Journal of Geophysical Research: Atmospheres*, *112*(D24), n/a-n/a. 10.1029/2007JD009050

Li, S.-H., Sun, J., & Li, B. (2011), Holocene environmental changes in central Inner Mongolia revealed by luminescence dating of sediments from the Sala Us River valley, *The Holocene*, *22*(4), 397-404. 10.1177/0959683611425543

Li, Y., Wang, N., Zhang, C., & Wang, Y. (2014), Early Holocene environment at a key location of the northwest boundary of the Asian summer monsoon: a synthesis on chronologies of Zhuye Lake, Northwest China, *Journal of Arid Land*, *6*(5), 511-528. 10.1007/s40333-014-0064-y

Li, Y., Wang, N., & Zhang, C. (2014), An abrupt centennial-scale drought event and mid-Holocene climate change patterns in monsoon marginal zones of East Asia. *PLoS ONE* 9, e90241. doi:10.1371/journal.pone.0090241.

Long, H., Lai, Z., Frenzel, P., Fuchs, M., & Haberzettl, T. (2012), Holocene moist period recorded by the chronostratigraphy of a lake sedimentary sequence from Lake Tangra Yumco on the south Tibetan Plateau, *Quaternary Geochronology*, *10*, 136-142. doi:10.1016/j.quageo.2011.11.005

Madsen, D.B., Haizhou, M., Rhode, D., Brantingham, P.J., & Forman, S.L. (2008), Age constraints on the late Quaternary evolution of Qinghai Lake, Tibetan Plateau, *Quaternary Research*, *69*(2), 316-325. doi:10.1016/j.yqres.2007.10.

Mathis, M., Sorrel, P., Klotz, S., & Oberhänsli, H. (2012), Holocene climate variability in lake Sonkul sediments (Kyrgyzstan, Central Asia) based on vegetation changes, *Geophys. Res. Abstracts*, 14, EGU2012-5523.

Mingram, J., Schettler, G., Nowaczyk, N., Luo, X., Lu, H., Liu, J., & Negendank, J.F.W. (2004), The Huguang maar lake—a high-resolution record of palaeoenvironmental and palaeoclimatic changes over the last 78,000 years from South China, *Quaternary International*, *122*(1), 85-107. doi:10.1016/j.quaint.2004.02.001

Morinaga, H., Itota, C., Isezaki, N., Goto, H., Yaskawa, K., Kusakabe, M., ... Cong, S. (1993), Oxygen-18 and carbon-13 records for the last 14,000 years from Lacustrine carbonates of Siling-Co (Lake) in the Qinghai-Tibetan Plateau, *Geophysical Research Letters*, *20*(24), 2909-2912. 10.1029/93GL02982

Morrill, C., Overpeck, J.T., Cole, J.E., Liu, K.-b., Shen, C., & Tang, L. (2006), Holocene variations in the Asian monsoon inferred from the geochemistry of lake sediments in central Tibet, *Quaternary Research*, *65*(2), 232-243. doi:10.1016/j.yqres.2005.02.014

Pan, B., Yi, C., Jiang, T., Dong, G., Hu, G., & Jin, Y. (2012), Holocene lake-level changes of Linggo Co in central Tibet, *Quaternary Geochronology*, *10*, 117-122. doi:10.1016/j.quageo.2012.03.009

Peck, J.A., Khosbayar, P., Fowell, S.J., Pearce, R.B., Ariunbileg, S., Hansen, B.C.S., & Soninkhishig, N. (2002), Mid to Late Holocene climate change in north central Mongolia as recorded in the sediments of Lake Telmen, *Palaeogeography, Palaeoclimatology, Palaeoecology*, *183*(1), 135-153. doi:10.1016/S0031-0182(01)00465-5

Penny, D., Grindrod, J., Bishop, P. (1996), Holocene palaeoenvironmental reconstruction based on microfossil analysis of a lake sediment core, Nong Han Kumphawapi, Udon Thani, Northeast Thailand, *Asian Persp.*, 35, 209-228.

Prokopenko, A.A., Khursevich, G.K., Bezrukova, E.V., Kuzmin, M.I., Boes, X., Williams, D.F., ... Abzaeva, A.A. (2007), Paleoenvironmental proxy records from Lake Hovsgol, Mongolia, and a synthesis of Holocene climate change in the Lake Baikal watershed, *Quaternary Research*, *68*(1), 2-17. doi:10.1016/j.yqres.2007.03.008

Rades, E.F., Tsukamoto, S., Frechen, M., Xu, Q., & Ding, L. (2015), A lake-level chronology based on feldspar luminescence dating of beach ridges at Tangra Yum Co (southern Tibet), *Quaternary Research*, *83*(3), 469-478. doi:10.1016/j.yqres.2015.03.002.

Ricketts, R.D., Johnson, T.C., Brown, E.T., Rasmussen, K.A., & Romanovsky, V.V. (2001), The Holocene paleolimnology of Lake Issyk-Kul, Kyrgyzstan: trace element and stable isotope composition of ostracodes, *Palaeogeography, Palaeoclimatology, Palaeoecology*, *176*(1), 207-227. doi:10.1016/S0031-0182(01)00339-X

Schwanghart, W., Schütt, B., & Walther, M. (2008), Holocene climate evolution of the Ugii Nuur basin, Mongolia, *Advances in Atmospheric Sciences*, *25*(6), 986-998. 10.1007/s00376-008-0986-4

Shen, J., Yang, L., Yang, X., Matsumoto, R., Tong, G., Zhu, Y., ... Wang, S. (2005), Lake sediment records on climate change and human activities since the Holocene in Erhai catchment, Yunnan Province, China, *Science in China Series D: Earth Sciences*, *48*(3), 353-363. 10.1360/03yd0118

Sheng, M., Wang, X., Zhang, S., Chu, G., Su, Y., & Yang, Z. (2017), A 20,000-year high-resolution pollen record from Huguangyan Maar Lake in tropical–subtropical South China, *Palaeogeography, Palaeoclimatology, Palaeoecology*, *472*, 83-92. doi:10.1016/j.palaeo.2017.01.038

Wang, H., Dong, H., Zhang, C.L., Jiang, H., Liu, Z., Zhao, M., & Liu, W. (2017), Deglacial and Holocene Archaeal Lipid-Inferred Paleohydrology and Paleotemperature History of Lake Qinghai, Northeastern Qinghai–Tibetan Plateau, *Quaternary Research*, *83*(1), 116-126. 10.1016/j.yqres.2014.10.003

Wang, S.M., and L. Ji (1995), Deposits and history of lake level fluctuation in Hulun Lake, J. *Lake Sci.*, 7, 297–308 (in Chinese).

Wünnemann, B., Chen, F., Riedel, F., Zhang, C., Mischke, S., Chen, G., ... Ming, J. (2003), Holocene lake deposits of Bosten Lake, southern Xinjiang, China, *Chinese Science Bulletin*, *48*(14), 1429-1432. 10.1360/02wd0270

Wünnemann, B., Mischke, S., & Chen, F. (2006), A Holocene sedimentary record from Bosten Lake, China, *Palaeogeography, Palaeoclimatology, Palaeoecology*, *234*(2), 223-238. doi:10.1016/j.palaeo.2005.10.016

Xiao, J., Si, B., Zhai, D., Itoh, S., & Lomtatidze, Z. (2008), Hydrology of Dali Lake in central-eastern Inner Mongolia and Holocene East Asian monsoon variability, *Journal of Paleolimnology*, *40*(1), 519-528. 10.1007/s10933-007-9179-x

Yang, X., Scuderi, L.A., Wang, X., Scuderi, L.J., Zhang, D., Li, H., ... Yang, S. (2015), Groundwater sapping as the cause of irreversible desertification of Hunshandake Sandy Lands, Inner Mongolia, northern China, *PNAS*, *112*(3), 702-706. 10.1073/pnas.1418090112

Zhang, H.C., Peng, J.L., Ma, Y.Z., Chen, G.J., Feng, Z.D., Li, B., ... Wünnemann, B. (2004), Late Quaternary palaeolake levels in Tengger Desert, NW China, *Palaeogeography, Palaeoclimatology, Palaeoecology*, *211*(1), 45-58.

Zhang, Z.Q., Wang, G.P., Lv, X.G., Jia, H.J., & Xu, Q.H. (2014), The sharp decline of East Asian summer monsoon at mid-Holocene indicated by the lake-wetland transition in the Sanjiang Plain, northeastern China, *Clim. Past Discuss.*, *2014*, 4595-4622. 10.5194/cpd-10-4595-2014

Zhao, Y., Yu, Z., Chen, F., Ito, E., & Zhao, C. (2007), Holocene vegetation and climate history at Hurleg Lake in the Qaidam Basin, northwest China, *Review of Palaeobotany and Palynology*, *145*(3), 275-288. doi:10.1016/j.revpalbo.2006.12.002

Zhao, C., Yu, Z., Zhao, Y., Ito, E., Kodama, K.P., & Chen, F. (2010), Holocene millennial-scale climate variations documented by multiple lake-level proxies in sediment cores from Hurleg Lake, Northwest China, *Journal of Paleolimnology*, *44*(4), 995-1008. 10.1007/s10933-010-9469-6

Zhao, C., Liu, Z., Rohling, E.J., Yu, Z., Liu, W., He, Y., ... Chen, F. (2013), Holocene temperature fluctuations in the northern Tibetan Plateau, *Quaternary Research*, *80*(1), 55-65. doi:10.1016/j.yqres.2013.05.001

Zhou, A, He, Y., Wu, D, Zhang, X, Zhang, C, Liu, Z, Yu, J. (2015) Changes in the Radiocarbon Reservoir Age in Lake Xingyun, Southwestern China during the Holocene. PLoS ONE 10(3): e0121532. https://doi.org/10.1371/journal.pone.0121532
